# SRHiC: A Deep Learning Model to Enhance the Resolution of Hi-C Data

**DOI:** 10.3389/fgene.2020.00353

**Published:** 2020-04-08

**Authors:** Zhilan Li, Zhiming Dai

**Affiliations:** ^1^School of Data and Computer Science, Sun Yat-sen University, Guangzhou, China; ^2^Guangdong Province Key Laboratory of Big Data Analysis and Processing, Sun Yat-sen University, Guangzhou, China

**Keywords:** chromatin three-dimensional structure, convolutional neural network, Hi-C data, super-resolution technology, human

## Abstract

Hi-C data is important for studying chromatin three-dimensional structure. However, the resolution of most existing Hi-C data is generally coarse due to sequencing cost. Therefore, it will be helpful if we can predict high-resolution Hi-C data from low-coverage sequencing data. Here we developed a novel and simple computational method based on deep learning named super-resolution Hi-C (SRHiC) to enhance the resolution of Hi-C data. We verified SRHiC on Hi-C data in human cell line. We also evaluated the generalization power of SRHiC by enhancing Hi-C data resolution in other human and mouse cell types. Results showed that SRHiC outperforms the state-of-the-art methods in accuracy of prediction.

## Introduction

Chromatin three-dimensional (3D) structure is vital to biological processes (Cremer and Cremer, 2001; Bonev and Cavalli, 2016), such as genome replication, DNA mutation and repair, transcription and so on. The advent of the high-throughput chromosome conformation capture (Hi-C) technique makes it possible to measure all pair-wise interactions across the entire genome (Lieberman-Aiden et al., 2009). The emergence and development of Hi-C technique have facilitated several exciting discoveries of A/B compartment (Lieberman-Aiden et al., 2009), topological associating domains (TADs) (Dixon et al., 2012), chromatin loops (Rao et al., 2014), and frequently interacting regions (FIREs) (Schmitt et al., 2016).

High-throughput chromosome conformation capture data is usually represented as a contact matrix *M*_*n* × *n*_, where *M*_*i,j*_ indicates the number of observed interactions (read pair count) between genomic regions *i* and *j*. The whole genome is partitioned into n fixed-size bins. Each bin corresponds to each row or each column in the matrix. The size (e.g., 10 Kb) of each bin is called the resolution of Hi-C contact matrix. Hi-C low-resolution data can be used to study A/B compartment or TAD, and Hi-C high-resolution data can be used to explore more elaborate structure (e.g., chromatin loop). Hi-C high-resolution data can offer deep insights into chromatin 3D structure. In general, high Hi-C sequencing coverage corresponds to high resolution of contact matrix. However, the linear increase of resolution requires a quadratic increase in the total number of sequencing reads. To address this issue, it is necessary to develop a computational method to predict high-resolution Hi-C contact maps from low-resolution Hi-C data.

In the past years, deep learning has achieved great achievement in many fields, including computer vision and natural language processing. Convolutional neural network (CNN) is a feedforward neural network (see Supplementary Material for background of deep learning). In biology, CNN have been applied in multiple subjects, such as predicting gene expression (Chen et al., 2016), prediction of DNA sequence function (Zhou et al., 2018), ploy-A site prediction and so on (Ma et al., 2014; Zhang et al., 2015; Pavlovic et al., 2017; Zeng and Gifford, 2017). Some pioneering studies have used CNNs to predict high-resolution Hi-C contact matrix from low-resolution Hi-C data. The Hi-C matrix was regarded as a single-channel picture, which can be simply understood as a gray-scale picture and can be processed using single-image super-resolution (SISR) technology. HiCPlus (Zhang et al., 2018) is the first work that applies a CNN, in which network architecture is similar to SRCNN (Dong et al., 2014), to enhance the resolution of Hi-C data. HiCNN (Liu and Wang, 2019), which was based on DRRN (Tai et al., 2017), used a very deep convolutional neural with 54 layers to predict the high-resolution Hi-C contact matrix from the low-resolution Hi-C contact matrix. HiCNN showed better prediction accuracy than HiCPlus, but the computational cost of HiCNN is much higher than that of HiCPlus.

In this study, we developed a novel method super-resolution Hi-C (SRHiC) based on the ResNet model (He et al., 2016) and the WDSR model (Yu et al., 2018). In our network, we improved the Res-block in ResNet to increase the nonlinearity of the network and improve the learning ability of the network. Meanwhile, we used a small convolution kernel multiple times to reduce the contact matrix size instead of using a big convolution kernel once. Our model outperformed HiCNN in prediction accuracy and running time, and outperformed HiCPlus in prediction accuracy with a slightly longer running time.

## Method

### Hi-C Data Preprocessing and Contact Matrix Generation

The data sets we used were from Gene Expression Omnibus (GEO) dataset under accession number GSE63525 in which Rao et al. (2014) provided high-resolution Hi-C paired-end reads in eight different cell types. To be consistent with datasets and experimental design in previous studies (Zhang et al., 2018; Liu and Wang, 2019), we used the combined contact matrix data of three of the eight cell types, including GM12878 (human), K562 (human), and CH12-LX (mouse). We chose the resolution 10 Kb as the high-resolution data for training and evaluation. The low-resolution data was produced by using a random down-sampling method to simulate the low-resolution Hi-C matrix as in previous studies (Zhang et al., 2018; Liu and Wang, 2019). In brief, we randomly selected part of the paired-end reads by the down sampling ratio (e.g., 1/16), and generated a low-resolution Hi-C contact matrix using the selected Hi-C paired-end reads. In order to generate the corresponding down-sampling data, we used two down-sampling ratios, 1/16 and 1/25. The former was to match the experimental design of the previous studies (Zhang et al., 2018; Liu and Wang, 2019), and the latter was to investigate whether the model can also have the same significant enhancement for lower resolution Hi-C data. Considering that a too low down-sampling ratio results in too many missing values in Hi-C matrix, we selected a moderate down-sampling ratio of 1/25. In GM12878, we used two different down-sampling ratios, 1/16 and 1/25. In K562 and CH12-LX, we only used the down-sampling ratio 1/16. We first used the Hi-C data from GM12878 with down-sampling ratio 1/16 and corresponding GM12878 high-resolution data to train a model which can enhance the low-resolution data to the high-resolution data, then used this trained model and the data from K562 and CH12-LX to evaluate the generalization capabilities of the model across different cell types in the same or different species. We also used the data from GM12878 with down-sampling ratio 1/25 to examine whether data with lower down-sampling ratio can be enhanced by our method. After data processing, the total number of high-resolution GM12878 Hi-C sequencing reads is 2.521 billion, the number for data with down-sampling ratios 1/16 is 0.158 billion and the number for 1/25 is 0.101 billion. Similarly, the total number of high-resolution K562 Hi-C sequencing reads is 0.488 billion and the number for 1/16 is 0.031 billion. The total number of high-resolution CH12-LX Hi-C sequencing reads is 0.321 billion and the number for 1/16 is 0.020 billion.

In the SRHiC model training process, we first divided 22 chromosomes (excluding sex chromosomes) into five groups. The first three groups include four chromosomes in order, for example, the group 1 contains chromosomes 1, 2, 3, and 4. The latter two groups, respectively, include five chromosomes, for example, the group 5 includes chromosomes 18, 19, 20, 21, and 22. We trained the model five times. Each time we selected four of the five groups as the training set. For the remaining one group, we randomly selected one chromosome as the validation set, and the remaining chromosomes as the test set. The specific validation set and test set used in each model training process were shown in the Supplementary Table S1. In this way, we got five trained models based on GM12878 data. When testing the generalization performance of the model across different cell lines, we randomly selected one of the five models obtained above to conduct a generalization performance test, and the second model was selected. Dealing with the GM12878 data with down-sampling ratio 1/25 training process, we only trained one model. We used most chromosomes in the validation sets in the training of the five models above as test set. The test set was chromosomes 2, 11, 14, and 21. The validation set was chromosome 7. The other 17 chromosomes were used as training set.

In the processing of training data, due to the Hi-C contact matrix of one chromosome is very large, we subdivided the whole contact matrix into 40 × 40 sub-matrices in order as in previous studies (Zhang et al., 2018; Liu and Wang, 2019). Since segmenting a large picture into small pictures may cause discontinuity in image information, in order to maintain the continuity of the image information, the same overlapping processing as in previous studies was used here. If the two sub-matrices are adjacent in the large matrix, their boundaries overlap. Take the operation in the row direction for example, if the first sub-matrix covers the rows of [1, 40] and columns of [1, 40], then the second sub-matrix will cover the rows of [29, 68] and columns of [1, 40]. The operation in the column direction is the same as the row direction. We also divided the high-resolution matrix into sub-matrices, but its size is 28 × 28 and there is no overlap between adjacent sub-matrices. Take the operation in the row direction for example, if the first sub-matrix covers the rows of [7, 34] and columns of [7, 34], then the second sub-matrix will cover the rows of [35, 62] and columns of [7, 34]. For example, the input sub-matrix at rows [1, 40] and columns [29, 68] corresponds to rows and columns of [7, 34] and [35, 62] in the output matrix. The enhanced sub-matrices predicted from the model were spliced into a large matrix.

### Overview of SRHiC

**FIGURE 1:**
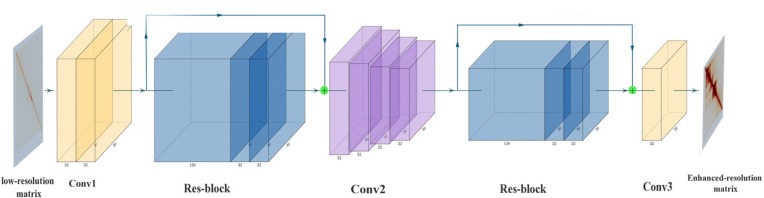
The architecture of SRHiC. It mainly consists of two Res-blocks and multiple convolutional layers. The input is the low-resolution Hi-C matrix, and the output is corresponding enhanced high-resolution Hi-C matrix output. The figure was generated by the tool PlotNeuralNet (https://github.com/HarisIqbal88/Plot NeuralNet.git).

Super-resolution Hi-C is based on CNN (Figure 1). SRHiC can enhance the low-resolution Hi-C contact matrix to the high-resolution Hi-C contact matrix. The input of the model is low-resolution sub-matrices with size of *n* × 40 × 40 × 1, where n represents the number of sub-matrices, and 1 indicates that the input is from single channel. We input these low-resolution sub-matrices into the model, SRHiC, and through enhanced by the model, we will get totally *n* × 28 × 28 × 1 high-resolution sub-matrice. In our network, Conv1 was mainly used to extract the features and patterns of the low-resolution Hi-C contact matrix from the input. As for Res-block, on one hand, the internal 1 × 1 convolution was used to increase the nonlinearity of the network, and on the other hand, the skip connection was used to allow the shallow information to flow directly to the deep network layer. The main role of Conv2 was to crop the size of the input Hi-C contact matrix. Conv2 used four small convolution kernels to crop the matrix four times. Conv3 was used as a prediction module to enhance the resolution of Hi-C data by using the features extracted from the previous network and to output high-resolution Hi-C contact matrix.

Conv1 contains two convolution layers, of which 2D convolution kernel sizes were 7 and 5, respectively, to extract the features from the input matrix. We have tested some larger 2D convolution kernel sizes (e.g., 13), but there was no improvement on the performance of the validation set. The number of feature map was 32. After extracting the features, a Res-block was used before cropping the matrix size. In Res-block, we first used the 1 × 1 convolution kernel (Lin et al., 2013) with the number of feature map as 128 to increase the depth of feature maps, which was followed by a rectified linear unit (ReLU) (Nair and Hinton, 2010). Then we used the 1 × 1 convolution kernel again with the number of feature map as 32 to reduce the depth of feature maps. We then used a convolution layer, of which 2D convolution kernel size was 7, with the number of feature map as 32 to extract the patterns of data. Finally, we added this result with previous input data into Res-block as the input of the subsequent network. In Res-block, we mainly used the 1 × 1 convolution to increase the nonlinearity of the network and improve the fitting ability of the network. This could help the deeper network layers to better learn the information of the shallow network layers. After the Res-block, we began to crop the matrix size by using the Conv2. We cropped the matrix size four times using a small convolution kernel size as cropping might lose margin information. The margins of the sub-matrices are not necessarily margin in the complete Hi-C contact matrix, therefore using a big convolution kernel size to crop the sub-matrix size at once might lose a lot of information. In the concrete network structure, the size of the 2D convolution kernel of the four crops was 5, 3, 5, and 3. The number of feature map of these four layers CNNs was 32 and each layer was followed by a ReLU. After cropping, we used the Res-block again mentioned above to prevent network degradation and deepen the depth of neural network. The predictive component of SRHiC included two convolutional layers, the Conv3 used a 7 × 7 convolutional kernel with the number of feature map as 32 to extract the pattern information and the second was to use a 5 × 5 convolutional kernel with the number of feature map as 1 to predict the target value by using the pattern information on neighboring values.

Given an input and target set ${\left\{X_{i},{\tilde{X}}_{i}\right\}}_{i=1}^{n}$, where *X*_*i*_ and ${\tilde{X}}_{i}$ are the low-resolution and corresponding high resolution Hi-C contact matrices. The loss function of SRHiC is

$$L\,\left(\Theta \right)=\frac{1}{n}\,\sum_{1}^{n}{∥F\,\left(X_{i}\right)-{\tilde{X}}_{i}∥}^{2}$$

where *F* was the mapping function SRHiC have learned to enhance the resolution of Hi-C contact matrix, and Θ represents the parameter space.

We implemented SRHiC with Tensorflow (v.1.13.1). The weight parameters of convolution kernel were initialized using the Xavier initialization method (Glorot and Bengio, 2010) and the optimizer was Adam (Kingma and Ba, 2014) with parameters initialized by default parameters and the batch size was 256. We used four NVIDIA V100 GPU with 64 Gb memory to train the model. The model was optimized by minimizing the mean square error (MSE) between the predicted (recovering from the low-resolution Hi-C data) and actual values (the corresponding high-resolution Hi-C data). The SRHiC source codes were available at https://github.com/hzlzldr/SRHiC. For HiCPlus and HiCNN, the python source codes were obtained from https://github.com/zhangyan32/HiCPlus and http://dna.cs.miami.edu/HiCNN/HiCNN_package.tar.gz, respectively. They were implemented by Pytorch. We used the Tensorflow (v.1.13.1) to reimplement them according to their parameters.

### Evaluation Methods

To quantitatively evaluate the performances of the three models, we used four metrics, including Pearson correlation coefficient, the stratum adjusted correlation coefficient (SCC) (Yang et al., 2017), statistically significant chromatin interactions and structural similarity index (SSIM) (Wang et al., 2004; see details in Supplementary Material).

## Results

### Recovering High-Resolution Hi-C Data From Low-Resolution Hi-C Data

**FIGURE 2:**
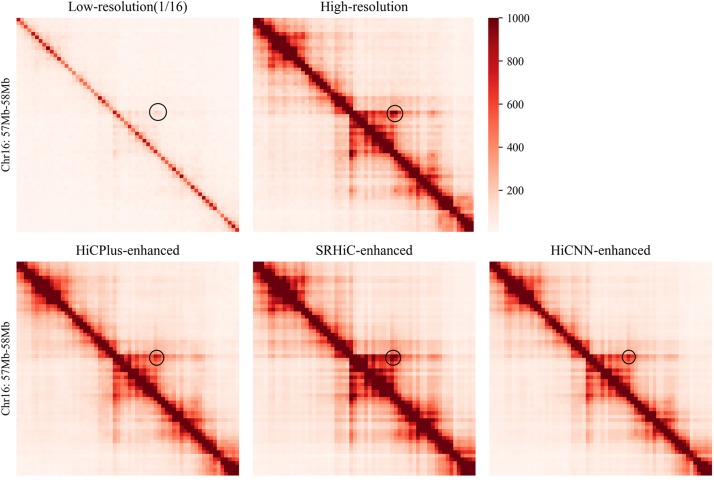
The Hi-C heat maps of chromosomes 16 (57–58 Mb) from five Hi-C data sets, including low-resolution with down sampling ratio 1/16, real high-resolution, HiCNN-enhanced, HiCPlus-enhanced, and SRHiC-enhanced.

We used the low-resolution data with down-sampling ratio 1/16 in GM12878. To provide a comprehensive evaluation, we trained five SRHiC models using different training sets (see details in section “Method” and Supplementary Table S1). For the two previous methods, HiCPlus and HiCNN, we also used similar strategy to train five models, respectively. We found that HiCNN showed much longer time for training than SRHiC and HiCPlus (Supplementary Table S1). In brief, the training time required by HiCNN was nearly 17.6 times that of HiCPlus, and the time required by SRHiC was 2.9 times that of HiCPlus. We applied the trained models to enhance the down-sampled Hi-C interaction matrix. Different models correspond to different training sets and test sets, and a total of 17 chromosomes were tested on the five trained models. We observed that the SRHiC-enhanced matrices are highly similar with the high-resolution Hi-C matrices (see an example in Figure 2).

**FIGURE 3:**
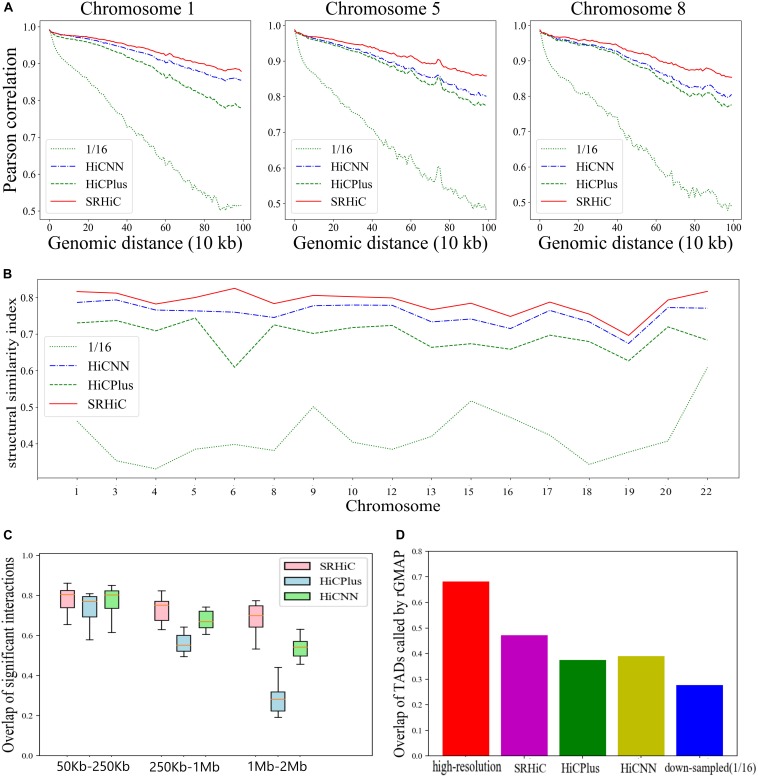
SRHiC can enhance chromatin interaction matrix with 1/16 down-sampling ratio in GM12878. **(A)** Pearson correlations between high-resolution Hi-C matrices and enhanced matrices predicted by the three models at each genomic distance in three example chromosomes. As the distance increases, the gap between SRHiC and the other two models becomes larger. **(B)** SSIM scores for the three models. **(C)** The overlap of chromatin interactions in a distance-range identified by the three methods with those from real high-resolution Hi-C data were shown for the 17 chromosomes included in the test sets. The source data were shown in Supplementary Table S2. **(D)** The TAD overlap between the original high-resolution Hi-C matrix and Hi-C inferred from the three models.

To quantitatively evaluate the performances of the three models, we calculated the Pearson correlation coefficient between the experimental high-resolution matrix and the matrices predicted by the three models at each genomic distance. We also calculated the Pearson correlation coefficient between the experimental high-resolution matrix and down-sampled matrix. The enhanced matrices by SRHiC showed higher correlation with experimental high-resolution matrices than those of HiCNN and HiCPlus (see three chromosome examples in Figure 3A, see the results of the other chromosomes in Supplementary Figure S1). Compared with the down-sampled Hi-C matrix, the enhanced Hi-C matrix showed significantly improved Pearson correlation coefficient score with the experimental high-resolution Hi-C matrix. As the distance increases, SRHiC showed better performance than the other two models. We used another metric, SCC score, to measure the similarity between the enhanced Hi-C matrix and the original high-resolution matrix. We used the HiCRep (Yang et al., 2017), a novel framework for assessing the reproducibility of Hi-C data that takes into account the unique spatial data features to calculate the SCC score. The results of the SCC score are highly consistent with the results of the Pearson correlation coefficient (Supplementary Figure S2). Compared with the down-sampling Hi-C matrix, the SCC scores of the Hi-C matrix enhanced by the three models are significantly improved. SRHiC showed higher SCC scores than HiCNN and HiCPlus. We also calculated the SSIM score to evaluate the performance of the three models. SRHiC had higher SSIM scores than HiCNN and HiCPlus (Figure 3B).

We next explored whether these enhanced high-resolution matrices can promote the identification of meaningful chromatin interactions and biological structures. We used Fit-Hi-C (Ay et al., 2014), which can identify statistically significant chromatin interactions by adjusting random polymer looping effect and estimating statistical confidence of intra-chromosomal interactions. Considering the average size of TADs is <1 Mb and there are few meaningful interactions outside TADs, we only restricted the analysis to chromatin interactions where the genomic distance between two loci is <2 Mb. The threshold for predicted significant interactions is *q*-value < 1e-06. We applied Fit-Hi-C to the real high-resolution (10 Kb) matrices, HiCPlus-enhanced interaction matrices, HiCNN-enhanced interaction matrices and SRHiC-enhanced interaction matrices in the 17 chromosomes included in the test sets. For each method, we calculated the overlap of its identified chromatin interactions with those identified from real high-resolution Hi-C data. We considered the overlap ratios in different distance-ranges, including 50–250 Kb, 250 Kb–1 Mb, 1 Mb–2 Mb (Figure 3C and Supplementary Table S2). In the 50–250 Kb interval, the overlap ratios of the three models are similar. However, as the interaction distance interval increases, the advantages of the SRHiC model become more obvious, while the performance of HiCPlus decreases significantly. In order to investigate whether the significant interaction pairs identified by the three models have biological significance, we downloaded relevant promoter-enhancer annotation data (Whalen et al., 2016). There are 159 annotated promoter-enhancer pairs identified by the original high-resolution Hi-C matrix. The three models recovered similar numbers of annotated promoter-enhancer pairs, ranging from 144 to 146 (Supplementary Table S3). Next, we used the original high-resolution Hi-C data as the standard to perform the Aggregate Peak Analysis (APA) (Rao et al., 2014) on the down-sampling Hi-C matrix and the enhanced Hi-C matrices to measure the aggregate enrichment of a set of putative peaks in a contact matrix. For quantitative comparison metric, we used P2LL score to indicate enrichment. SRHiC showed higher P2LL score than the other two models (Supplementary Figure S3).

We further tested whether the three model can recover annotated TAD boundaries from the TAD knowledge base (TADKB) (Liu and Wang, 2019). We used the Gaussian Mixture model And Proportion test (GMAP) (Yu et al., 2017) to identify TADs from the enhanced Hi-C matrices, low resolution and high resolution data Hi-C matrices. We defined contact overlapped boundary if it falls within 0.2 Mb of any annotation contact boundary. SRHiC could recover more TAD boundaries than HiCPlus and SRHiC (Figure 3D and Supplementary Figure S4).

We next sought to examine whether SRHiC can enhance chromatin interaction matrix with lower down-sampling ratio 1/25. We used the low-resolution data with down-sampling ratio 1/25 to train a new model for SRHiC, HiCNN, and HiCPlus, respectively (see details in section “Method” and Supplementary Table S1). We used the same evaluation metrics as above. We found that SRHiC still showed better performance than HiCNN and HiCPlus in all the four chromosomes included in the test sets (Supplementary Figures S5–S8 and Supplementary Table S4). However, as the number of sequencing reads decreases (see the method section “Hi-C Data Preprocessing and Contact Matrix Generation” for the total number of reads after different sampling ratios), all the three models showed decrease in performance. The reason is largely due to insufficient sequencing depth, which makes a large number of entries in the Hi-C matrix become value 0, significantly impacting on the training and learning of the models.

### Enhancing Hi-C Interaction Matrices Across Different Cell Types

**FIGURE 4:**
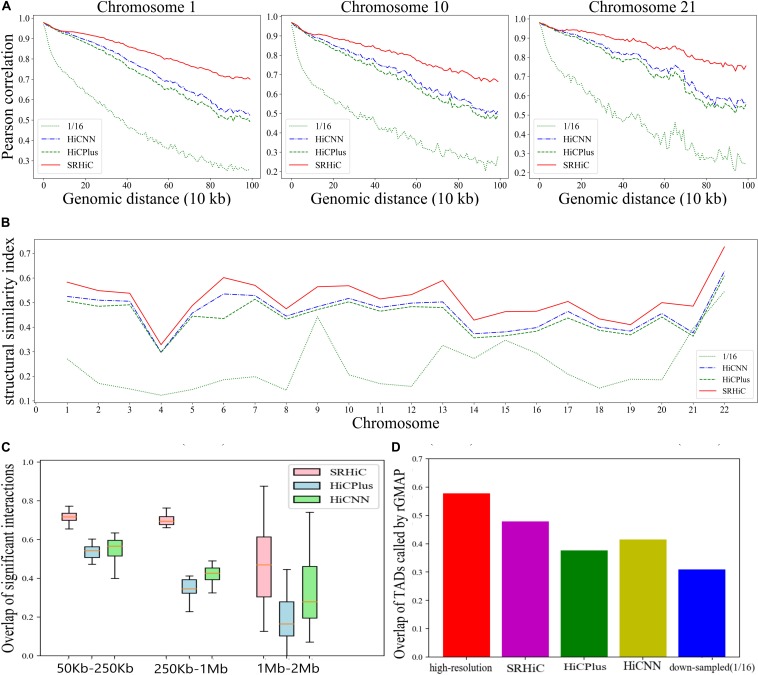
SRHiC can enhance chromatin interaction matrix across different cell types. The models trained on GM12878 dataset were used to enhance matrices in K562. **(A)** Pearson correlations between high-resolution Hi-C matrices and enhanced matrices predicted by the three models at each genomic distance in three example chromosomes. **(B)** SSIM scores for the three models. **(C)** The overlap of chromatin interactions in a distance-range identified by the three methods with those from real high-resolution Hi-C data were shown for the 22 chromosomes. The source data were shown in Supplementary Table S5. **(D)** The TAD overlap between the original high-resolution Hi-C matrix and Hi-C inferred from the three models.

We examined whether our model trained on one cell type can be directly used to enhance Hi-C matrices of other cell types in the same species. Firstly, we randomly selected one of the five models trained on GM12878 with down-sampling ratio 1/16 shown in Supplementary Table S1. We randomly selected the second model, in which the training set was chromosomes 1∼4 and 9∼22. Then, we used this trained model to enhance the Hi-C down-sampling (ratio 1/16) matrices of all 22 chromosomes in K562. Using the same evaluation strategies as above, we calculated the Pearson correlation coefficient and the SCC score between the experimental high-resolution matrix and the predicted matrices. We also examined the overlap of chromatin interactions from the predicted matrices with those from the real high-resolution matrix. The enhanced matrices by SRHiC showed higher correlation and SCC scores with experimental high-resolution matrices than those of HiCNN and HiCPlus in all 22 chromosomes (see the Pearson correlation coefficient of three chromosome examples in Figure 4A and the other chromosomes in Supplementary Figure S9, see the SCC score in Supplementary Figure S10). In terms of the SSIM score, overlap with real chromatin interactions and TAD recovery, SRHiC still maintains better performance than the other two models (Figures 4B–D, Supplementary Figures S11, S12, and Supplementary Tables S5, S6).

We examined whether our model trained on human cell type can be directly used to enhance Hi-C matrices of mouse cell type. We used the second model trained on human GM12878 shown in Supplementary Table S1, to enhance the Hi-C down-sampling (ratio 1/16) matrices of all 19 chromosomes in mouse CH12-LX cell type. The enhanced results from SRHiC showed a higher Pearson correlation, SCC score, overlap with distance-range and SSIM score with experimental high-resolution data than those from HiCNN and HiCPlus in all 19 chromosomes (Supplementary Figures S13–S17 and Supplementary Table S7).

## Discussion

Although our model used only a moderate number of network layers, it can successfully infer the corresponding high-resolution Hi-C interaction matrices from low-resolution ones with down-sampling ratios 1/16 and 1/25. This will help researchers acquire high-resolution Hi-C data with less sequencing cost. From these results we can see that when training and prediction are applied to the data of a cell line (such as GM12878), whether it is on the consistency score with the original high-resolution Hi-C matrix, or the identification of related biological structures, including the identification of significant interaction pairs and TAD boundary, SRHiC is better than the other two models. Especially when it comes to the identification of long-distance interaction pairs, SRHiC is far superior to HiCPlus and HiCNN. In the lower-resolution (1/25) training data experiment, we can see that the three models have more or less decreased on multiple metrics. However, this decline does not mask the enhanced Hi-C matrix inferred by SRHiC, which is closer to the original high-resolution Hi-C matrix than the two models. This conclusion can be drawn from relevant results such as Pearson correlation coefficient or APA analysis.

When comparing the generalization capabilities of the three models across cell lines (K562) and species (mouse CH12-LX), we found that they all showed a decline in performance compared with GM12878. However, SRHiC has a smaller decline than HiCNN and HiCPlus. HiCPlus is a simple three-layer neural network. Too few network layers might make the network incapable of learning the map function behind the data. Compared with HiCPlus, HiCNN network is very deep with 54 layers. Our results showed that HiCNN showed better performance than HiCPlus, but showed worse performance than SRHiC. SRHiC has fewer layers than HiCNN. HiCNN cropped the matrix size only at once. Considering that cropping may lose margin information, SRHiC cropped the matrix size four times using a small convolution kernel size. When a single sub-matrix is cropped, its margin is cropped. But the margins of the sub-matrices are not necessarily margin in the complete Hi-C contact matrix, therefore using a big convolution kernel size to crop the sub-matrix size at once might lose a lot of information. A too deep or complex network might be not a good choice, as it may have an over-fitting property that causes a relatively big decline in generalization capability.

Constrained by sequencing errors, the data that we used for model training and evaluation inevitably have noise. The more reliable the given data is, the more accurately the biological rules the model can learn. Therefore, the emergence of better sequencing technology will generate more reliable experimental data and make computational methods more accurate. It will be necessary to develop models to enhance low-resolution Hi-C data accounting for sequencing errors.

## Data Availability Statement

Publicly available datasets were analyzed in this study. This data can be found here: the Gene Expression Omnibus (GEO) accession number for the data sets used in this article is GSE63525.

## Author Contributions

ZL and ZD designed the study, analyzed the results, and drafted the manuscript. ZL implemented the algorithms and carried out the experiments.

## Conflict of Interest

The authors declare that the research was conducted in the absence of any commercial or financial relationships that could be construed as a potential conflict of interest. The reviewer VB and handling Editor declared their shared affiliation.
