# Supplementary material for: SRHiC: A Deep Learning Model to Enhance the Resolution of Hi-C Data
Source: Front Genet. 2020 Apr 8;11:353. doi: 10.3389/fgene.2020.00353 (PMC7156553; doi:10.3389/fgene.2020.00353)
Supplement: Supplementary file 1 [file Data_Sheet_1.PDF]

Supplementary Materials

SRHiC: A deep learning model to enhance the resolution of Hi-C data

Zhilan Li[1], Zhiming Dai[1,2§]

[1]School of Data and Computer Science, Sun Yat-Sen University, Guangzhou 510006, China
[2]Guangdong Province Key Laboratory of Big Data Analysis and Processing, Sun Yat-Sen University, Guangzhou 510006, China

[§]daizhim@mail.sysu.edu.cn

Supplementary text
Figures S1~S17
Tables S1~S7

Supplementary text

Background of deep learning

Basic structure of CNN consists of an input layer, multiple hidden layers and an output layer. The hidden layer generally includes multiple convolutional layers and multiple pooling layers. Convolutional layers and pooling layers are alternately arranged, that is, one convolutional layer is connected to one pooling layer. Deep convolutional neural networks have led to a series of breakthroughs for computer vision. However, with the network depth increasing, a degradation problem will be exposed. The accuracy will get saturated and then degrade rapidly. Obviously, such degradation is not caused by overfitting, and adding more layers to a suitably deep model lead to higher training error. Deep Residual Networks(ResNet) (He, et al., 2016) is a type of CNN. It introduces the technique of skip-connection to directly input shallow information into the deep network, so that the deep network layer can directly learn the shallow information of the network, which solves the problem to a certain extent of network degradation, laying the foundation for the development of deep neural networks. WDSR(Yu, et al., 2018), which is the winner of NTIRE2018 Super Resolution Competition, is a neural network model based on CNN. W here indicates wide, that is, it has a broader feature map, which can also be understood as more feature maps. WDSR shows to some extent that the use of wider feature maps between network layers will enhance the model's super-resolution capabilities. In the field of deep learning, the mainly application of recurrent neural networks (RNN) is to process sequence data. The information of the next unit is extremely dependent on the output of the previous unit. Therefore, the training time required for the RNN is much longer than that of the CNN with the same number of layers.

Evaluation methods

Pearson’s correlation coefficient is used to measure the linear correlation between the enhanced and ground truth high-resolution Hi-C data with the distance-range. At a specific distance value (abscissa value), the higher Pearson’s correlation score, the

more consistent between the predicted and real Hi-C data. Meanwhile, we used the SCC score in order to more highlight other features of the matrix, except the dependence on distance. This is a special metric for comparing Hi-C matrices, based on Pearson correlation. SSIM is used for measuring the similarity between computationally predicted and experimental Hi-C matrix. SSIM ranges from 0 to 1. The closer to 1, the higher similarity the two matrices have. SSIM is designed to improve on traditional methods such as peak signal-to-noise ratio (PSNR) and mean squared error (MSE).

Reference

He, K.M., *et al.* (2016) Deep Residual Learning for Image Recognition, *Proc Cvpr Ieee*, 770-778.

Yu, J., *et al.* (2018) Wide Activation for Efficient and Accurate Image Super-Resolution. *arXiv e-prints*.

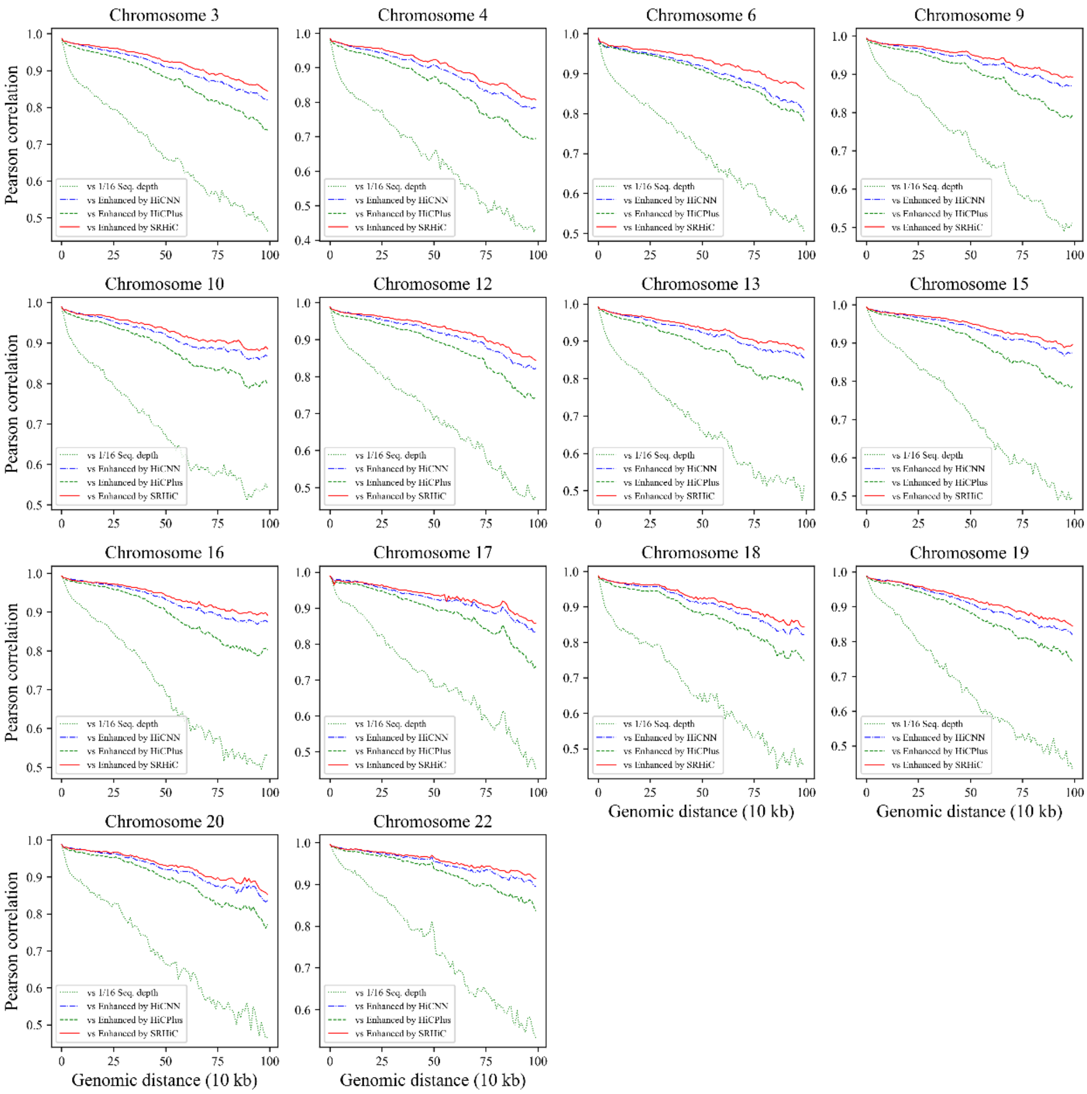

Figure S1. Same as Figure 3A, but for the remaining test chromosomes.

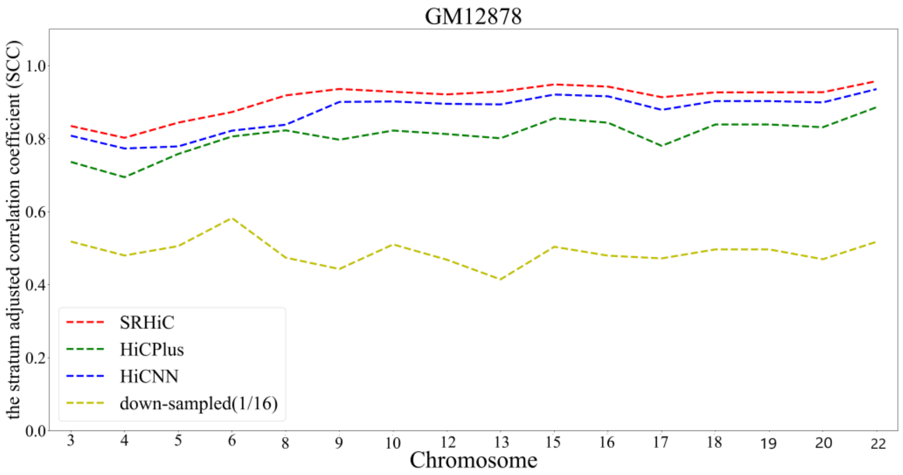

Figure S2. The SCC scores for each test chromosomes. The SCC score is mainly used to measure the reproducible of two Hi-C matrices. The value ranges from -1 to 1. The closer to 1, the higher the reproducible. Because chromosome 1 is too large for computer memory to calculate, it is missing. We can see from other chromosomes that the Hi-C matrix inferred by SRHiC has the highest SCC score.

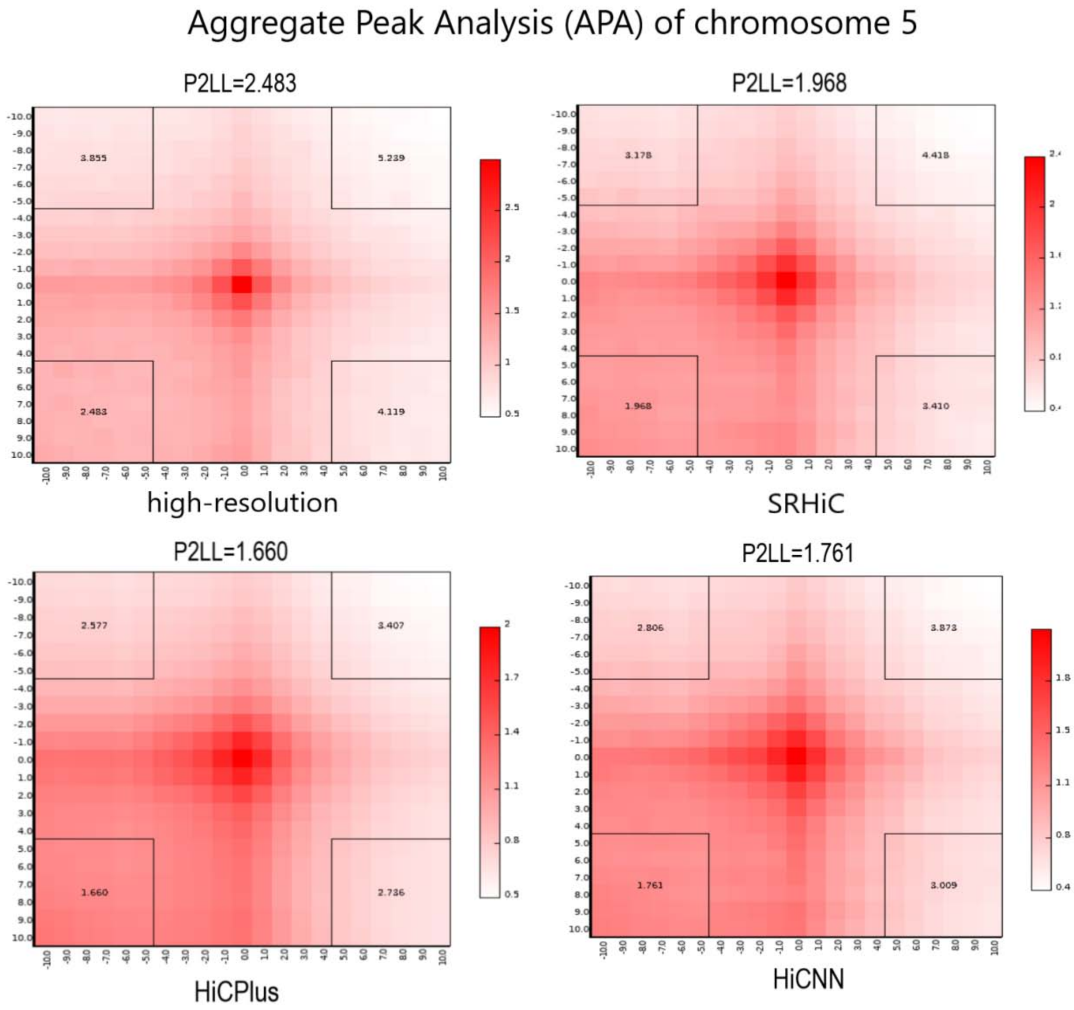

Figure S3. The Aggregate Peak Analysis (APA) of chromosome 5 in GM12878 with down-sampled (1/16). APA is mainly use to measure the aggregate enrichment of a set of putative peaks in a contact matrix. The P2LL (Peak to Lower Left) score is recommended to use for a quantitative comparison metric, which values significantly above 1 indicate enrichment. According to the calculation results, the P2LL of SRHiC is greater than 1 and higher than that obtained by the other three matrices.

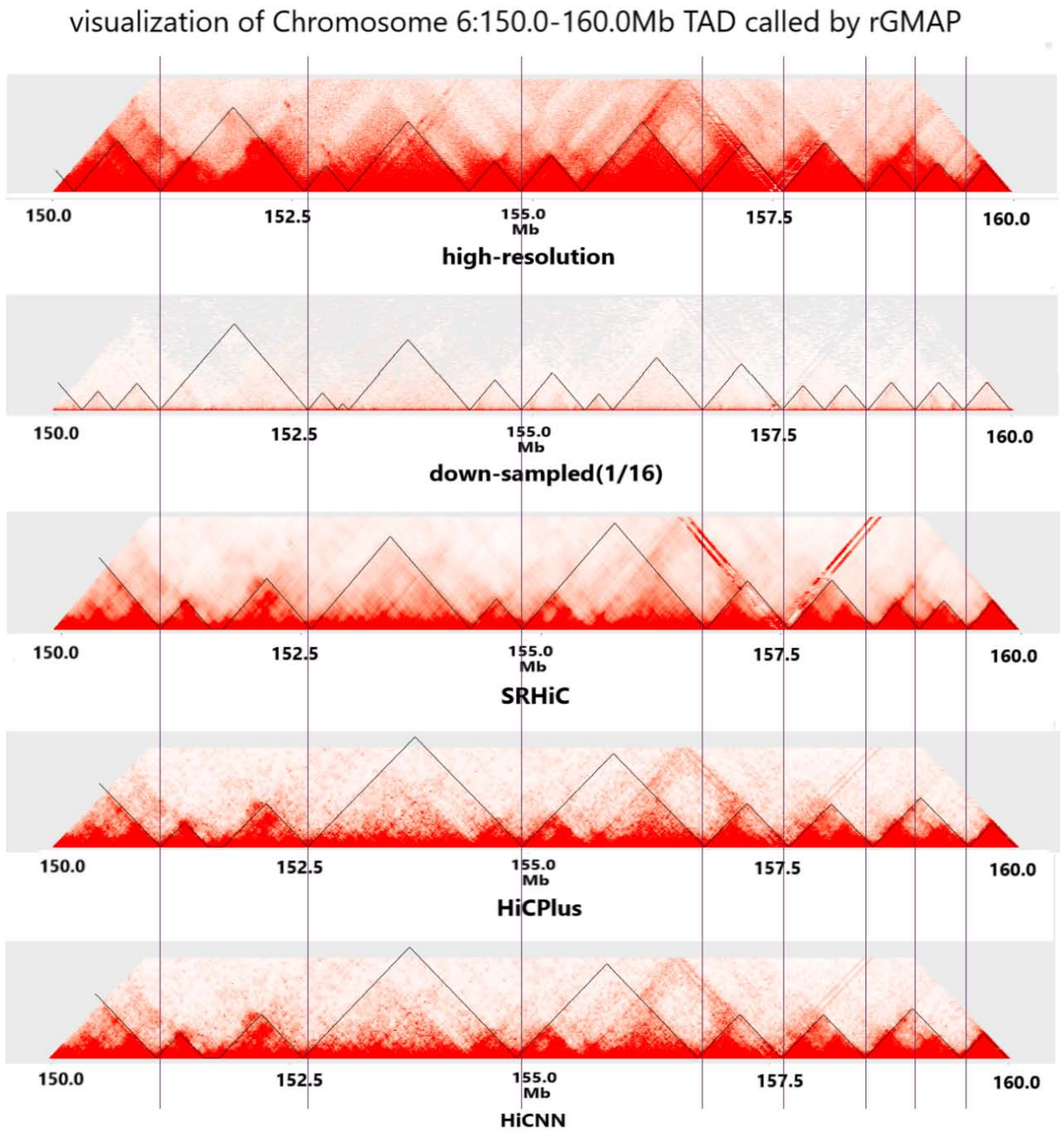

Figure S4. The TAD boundary visualization of Chromosome 6:160.0-160.0Mb with down-sampled (1/16) in GM12878. SRHiC has many coincident TAD boundaries with the original high-resolution Hi-C matrix called by rGMAP.

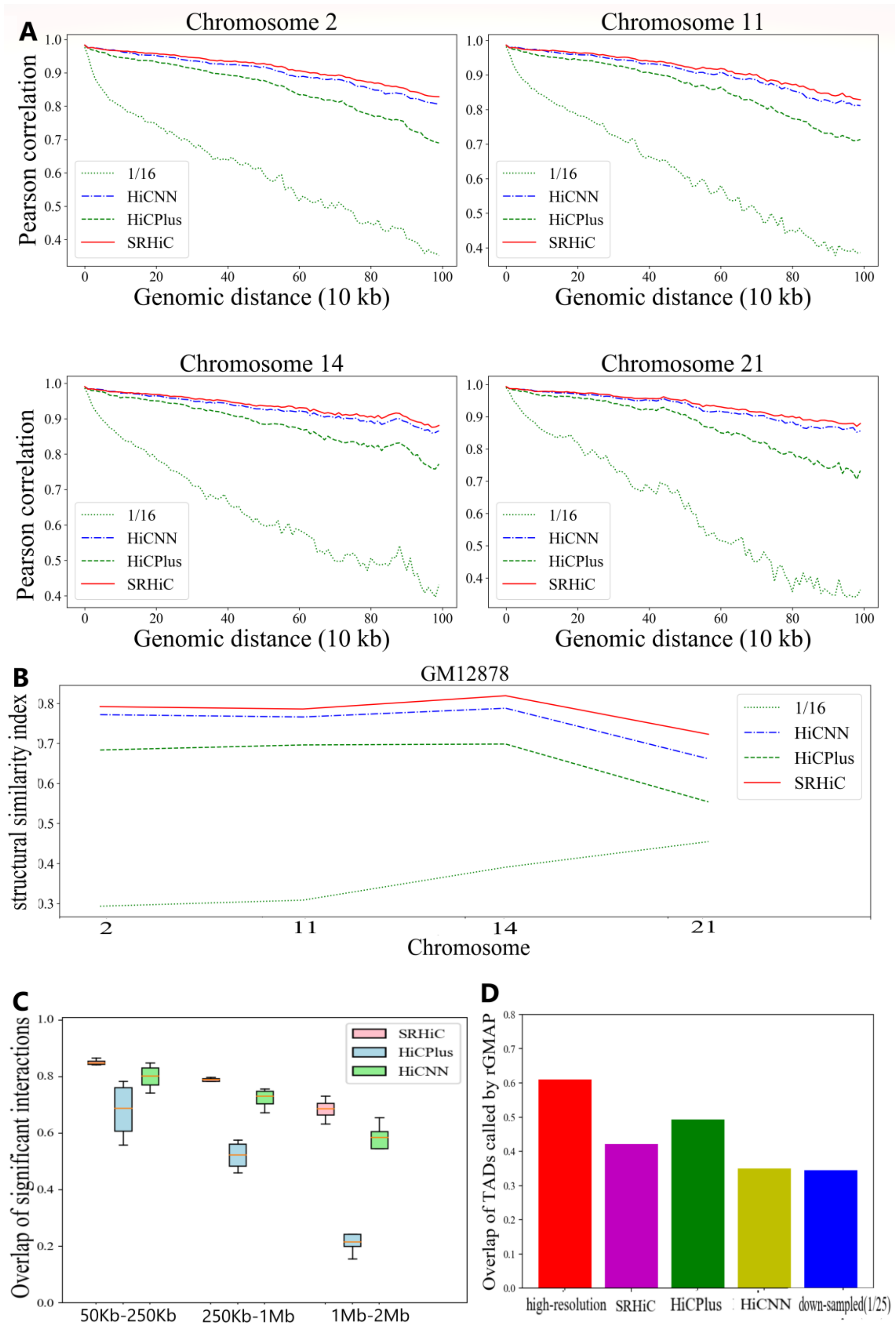

Figure S5. SRHiC can enhance chromatin interaction matrix with 1/25 down-sampling ratio in GM12878. Compared with down-sample (1/16) Hi-C data, the performance of several indicators has decreased, but SRHiC is still better than HiCPlus and HiCNN in performance. This decline in performance may be due to too low sequencing depth, which results in too many zero entries in the Hi-C matrix, which affects the training of the model. (A) Pearson correlations between high-resolution Hi-C matrices and enhanced matrices predicted by the three models at each genomic distance in the test chromosomes. (B) SSIM scores for the three models. (C) The overlap of chromatin interactions in a distance-range identified by the three

methods. The source data were shown in Table S4. (D) The TAD called by rGMAP from the original high-resolution Hi-C matrix and Hi-C inferred from the three models overlap with TADKB dataset.

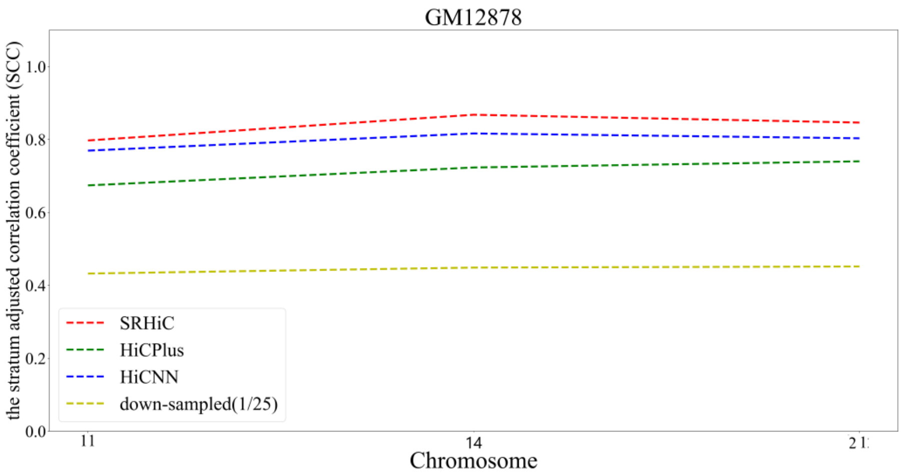

Figure S6. The SCC score for each test chromosomes with down-sampled (1/25) in GM12878.

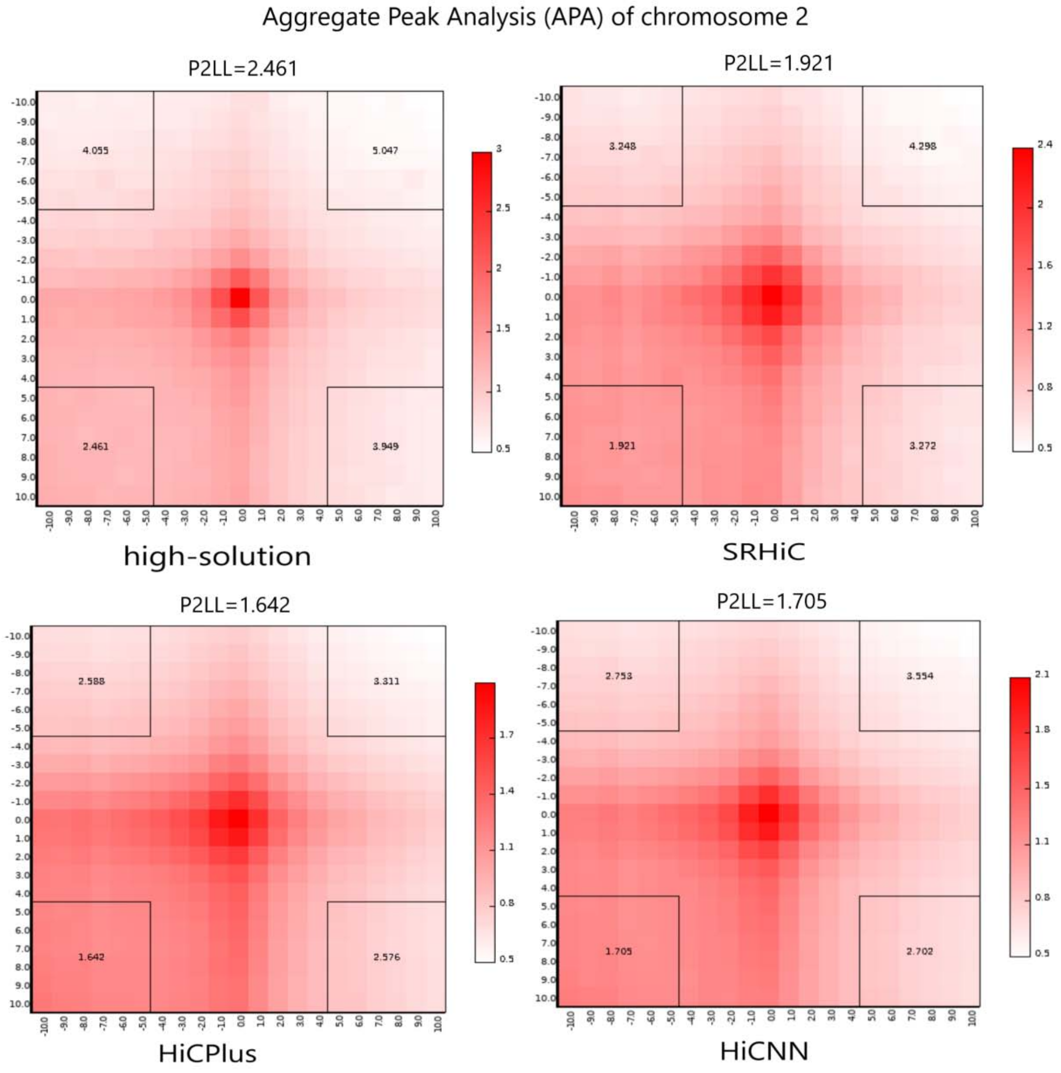

Figure S7. The Aggregate Peak Analysis (APA) of chromosome 2 in GM12878 with down-sampled (1/25).

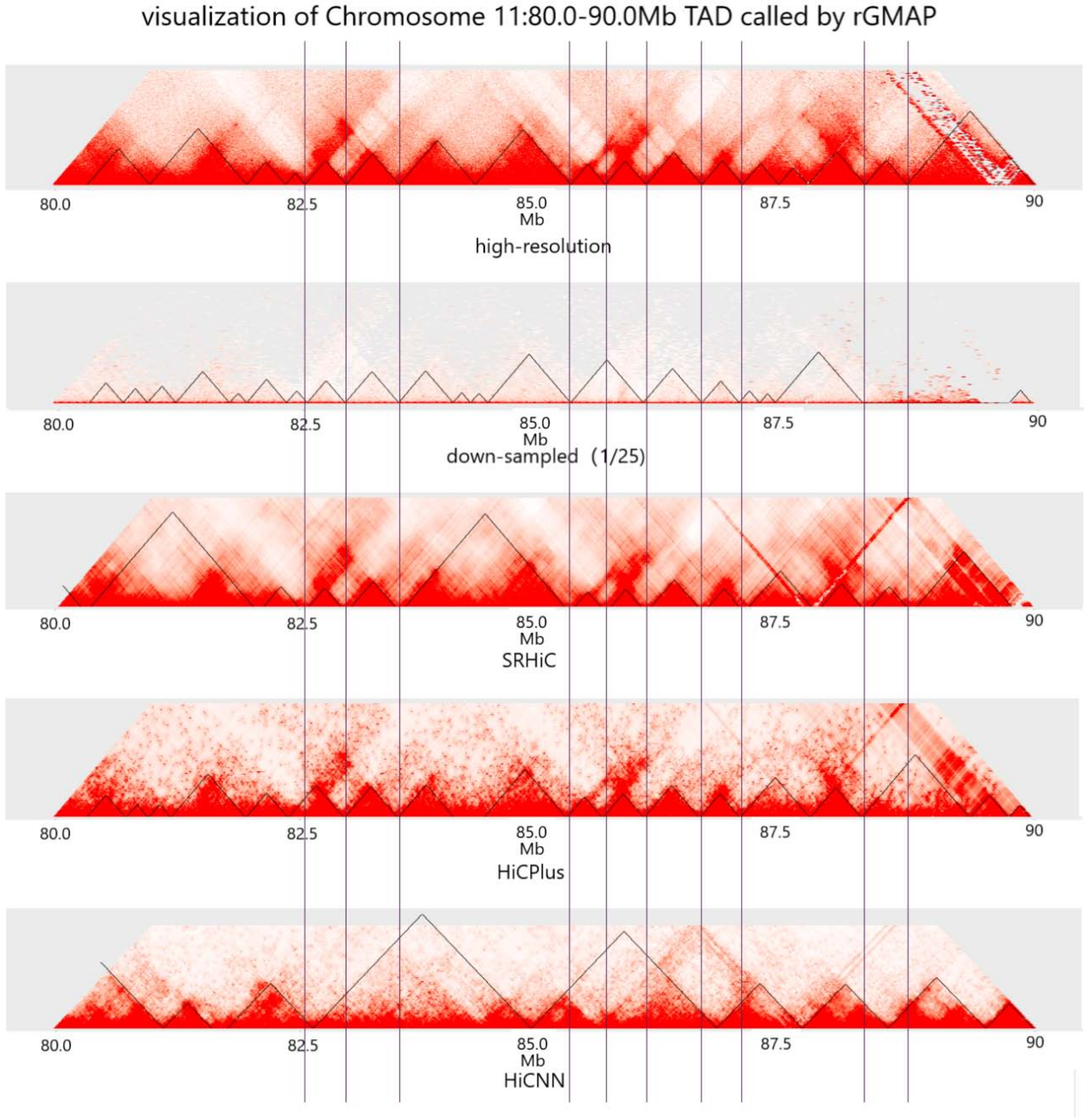

Figure S8. The TAD boundary visualization of Chromosome 11:80.0-90.0Mb with down-sampled (1/25) in GM12878.

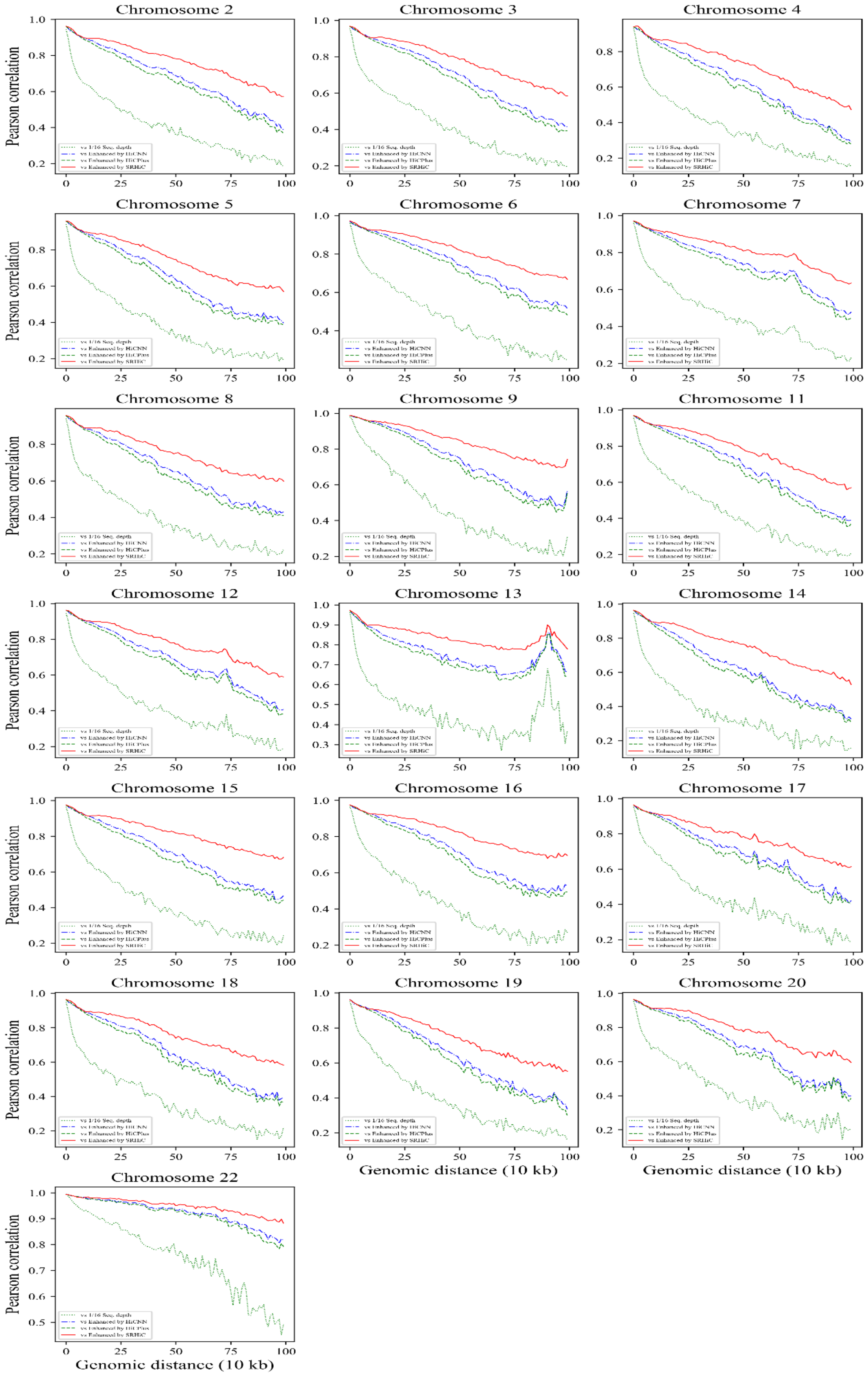

Figure S9. Same as Figure 4A, but for the remaining test chromosomes.

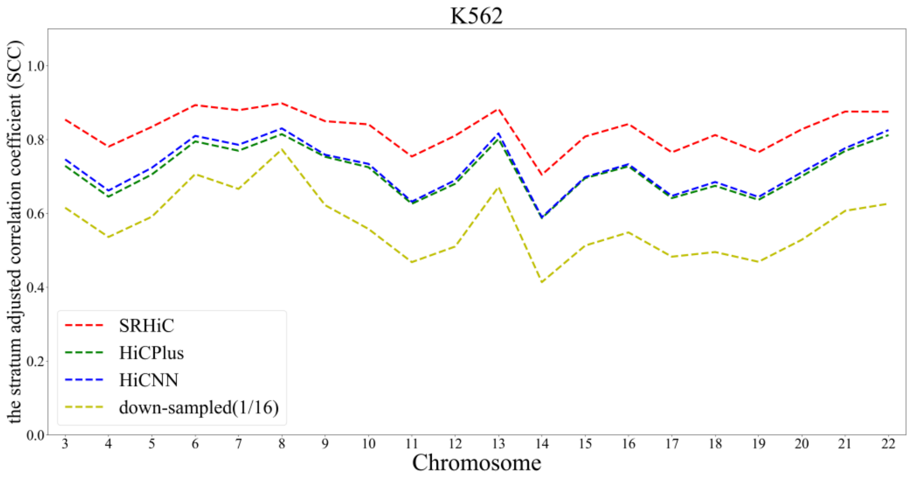

Figure S10. The SCC score for each test chromosomes with down-sampled (1/16) in K562. Because chromosome 1,2 is too large for computer memory to calculate, it is missing.

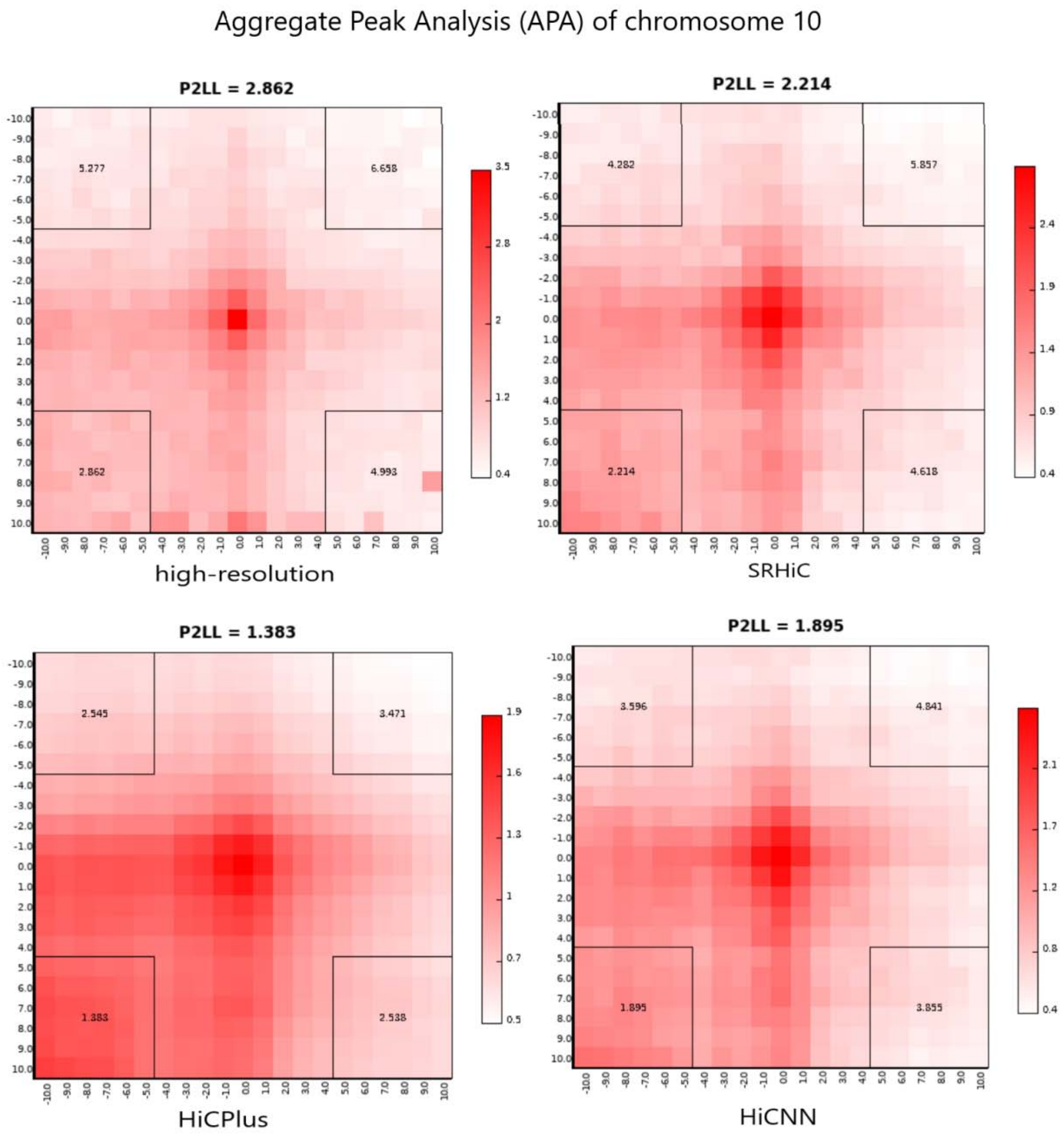

Figure S11. The Aggregate Peak Analysis (APA) of chromosome 10 in K562 with down-sampled (1/16).

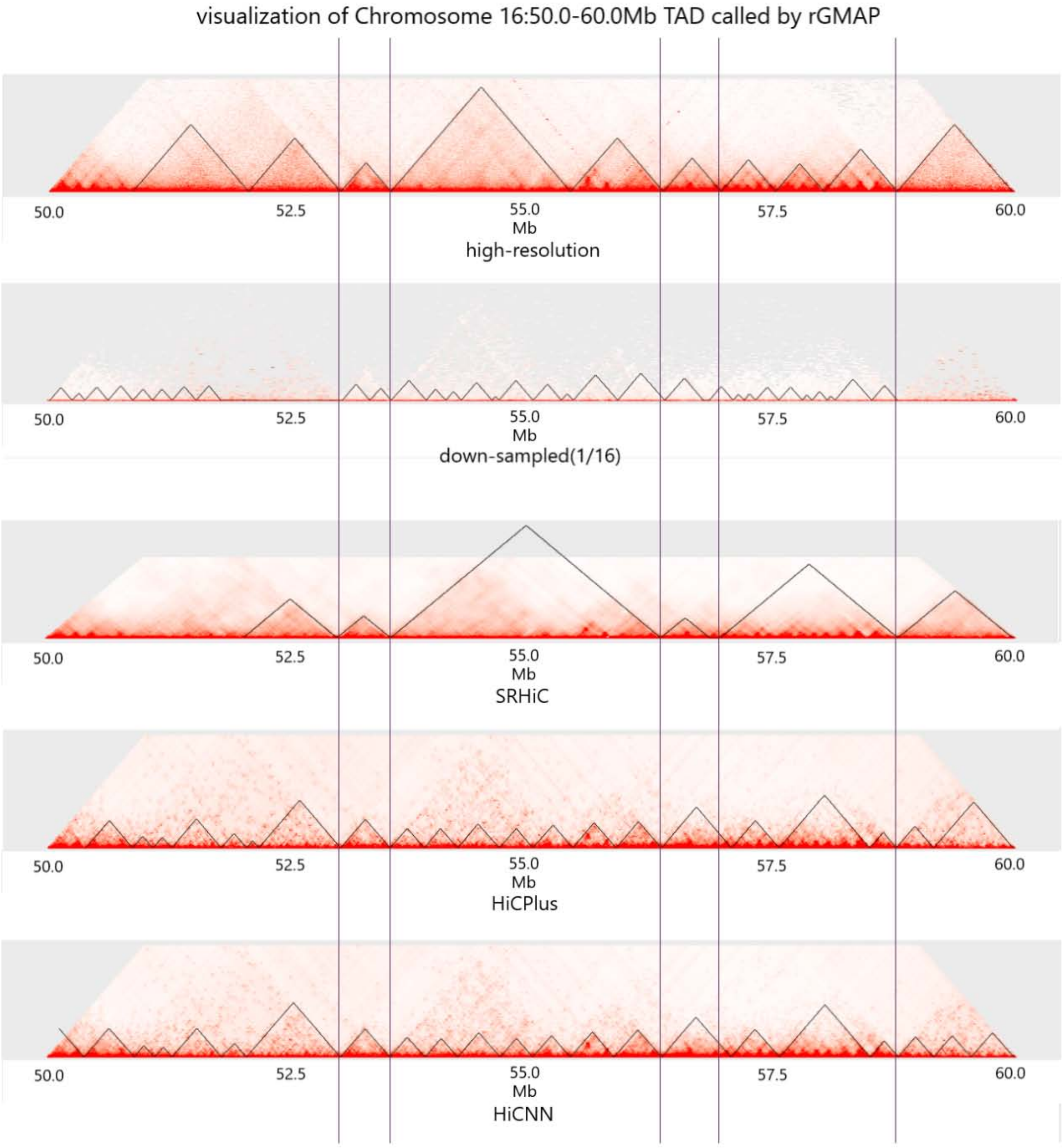

Figure S12. The TAD boundary visualization of Chromosome 16:50.0-60.0Mb with down-sampled (1/16) in K562.

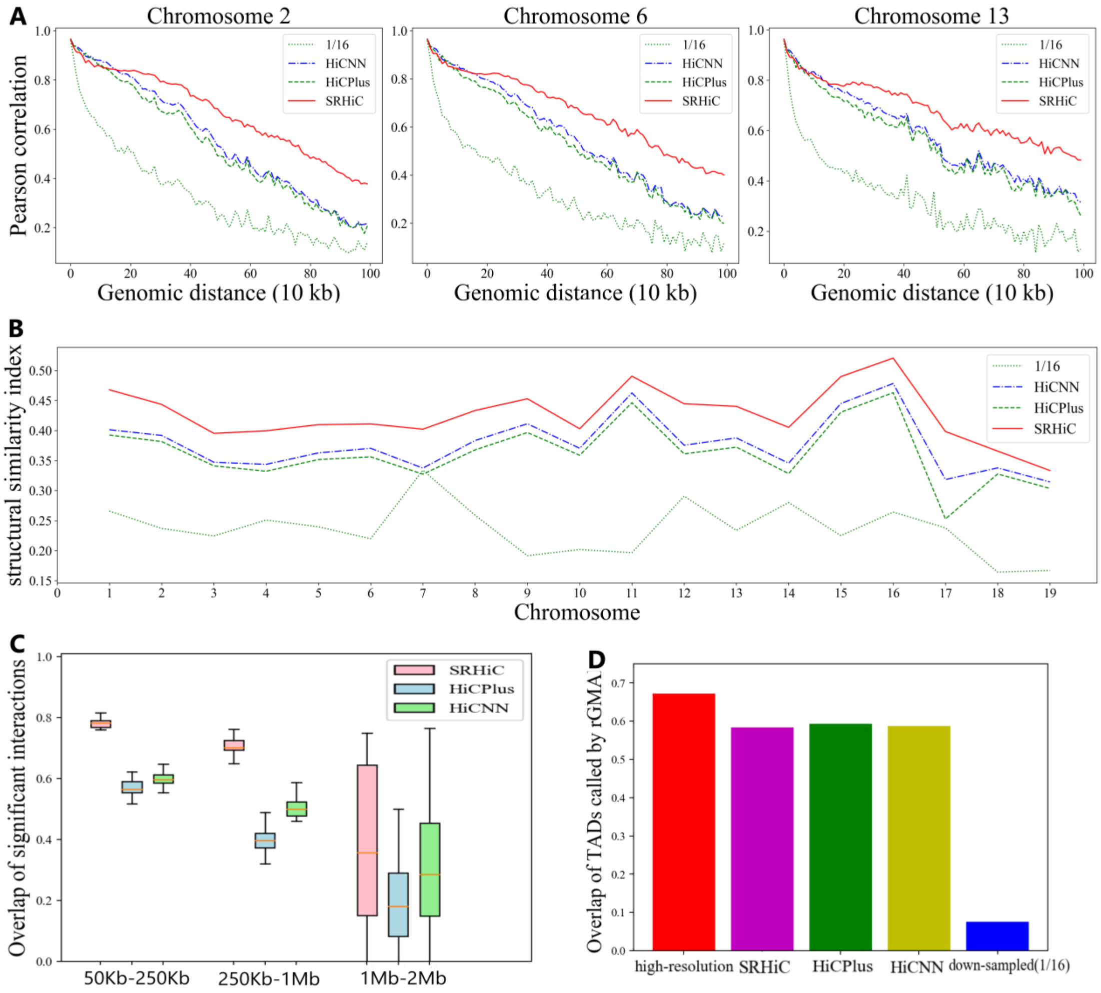

Figure S13. SRHiC can enhance chromatin interaction matrix across different species types. The models trained on GM12878 dataset were used to enhance matrices in CH12-LX. (A) Pearson correlations between high-resolution Hi-C matrices and enhanced matrices predicted by the three models at each genomic distance in three example chromosomes. (B) SSIM scores for the three models. (C) The overlap of chromatin interactions identified by the three methods with those from real high-resolution Hi-C data in a distance-range were shown for the 19 chromosomes. The source data were shown in Table S7. (D) The TAD called by rGMAP from the original high-resolution Hi-C matrix and Hi-C inferred from the three models overlap with TADKB dataset.

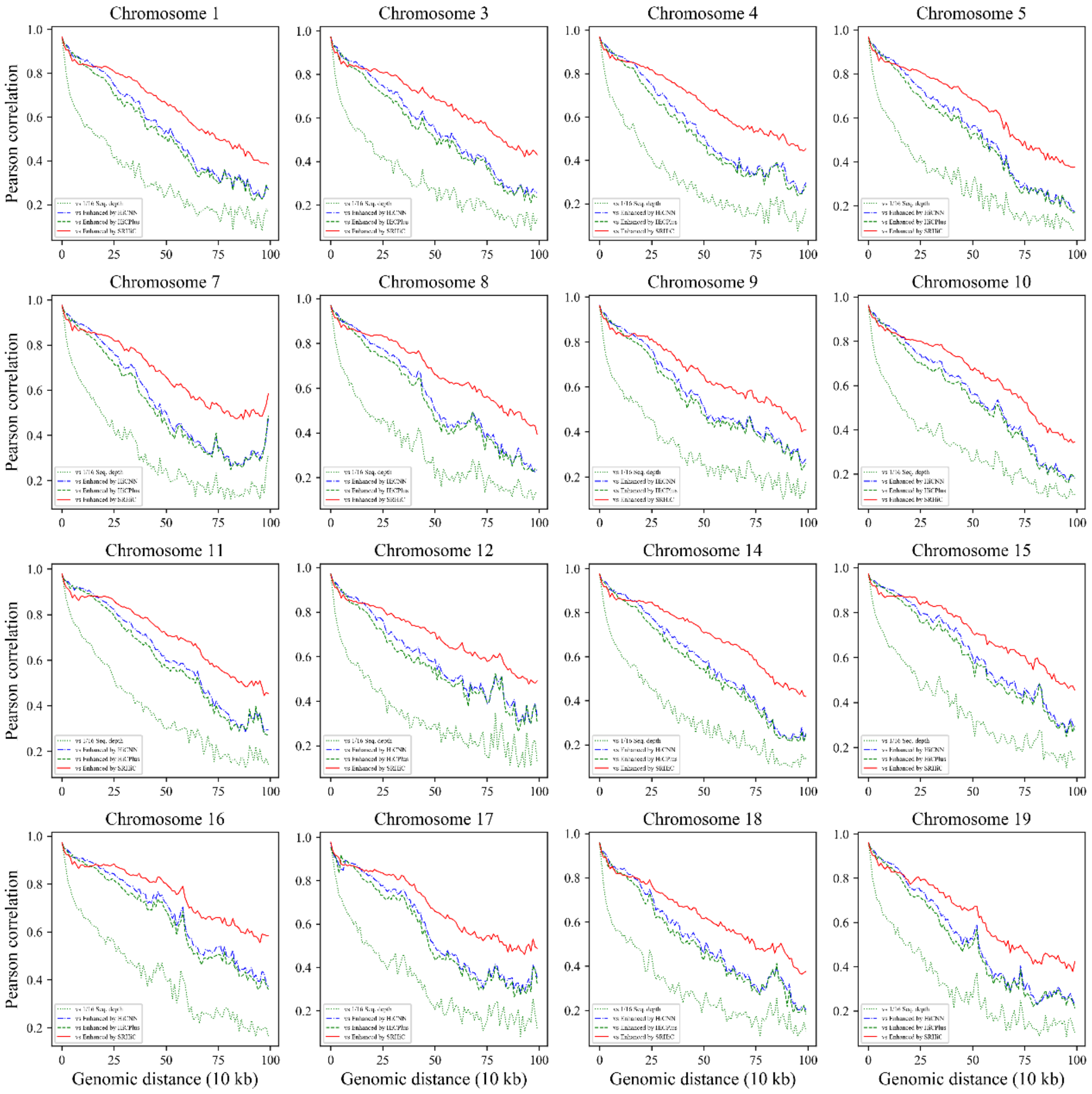

Figure S14. Same as Figure S13A, but for the remaining test chromosomes.

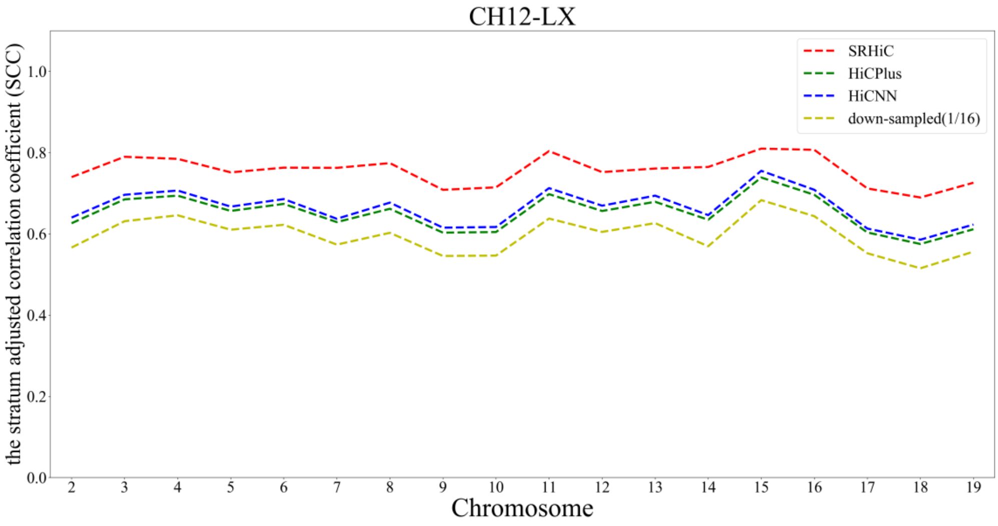

Figure S15. The SCC score for each test chromosomes with down-sampled (1/16) in CH12-LX. Because chromosome 1 is too large for computer memory to calculate, it is missing.

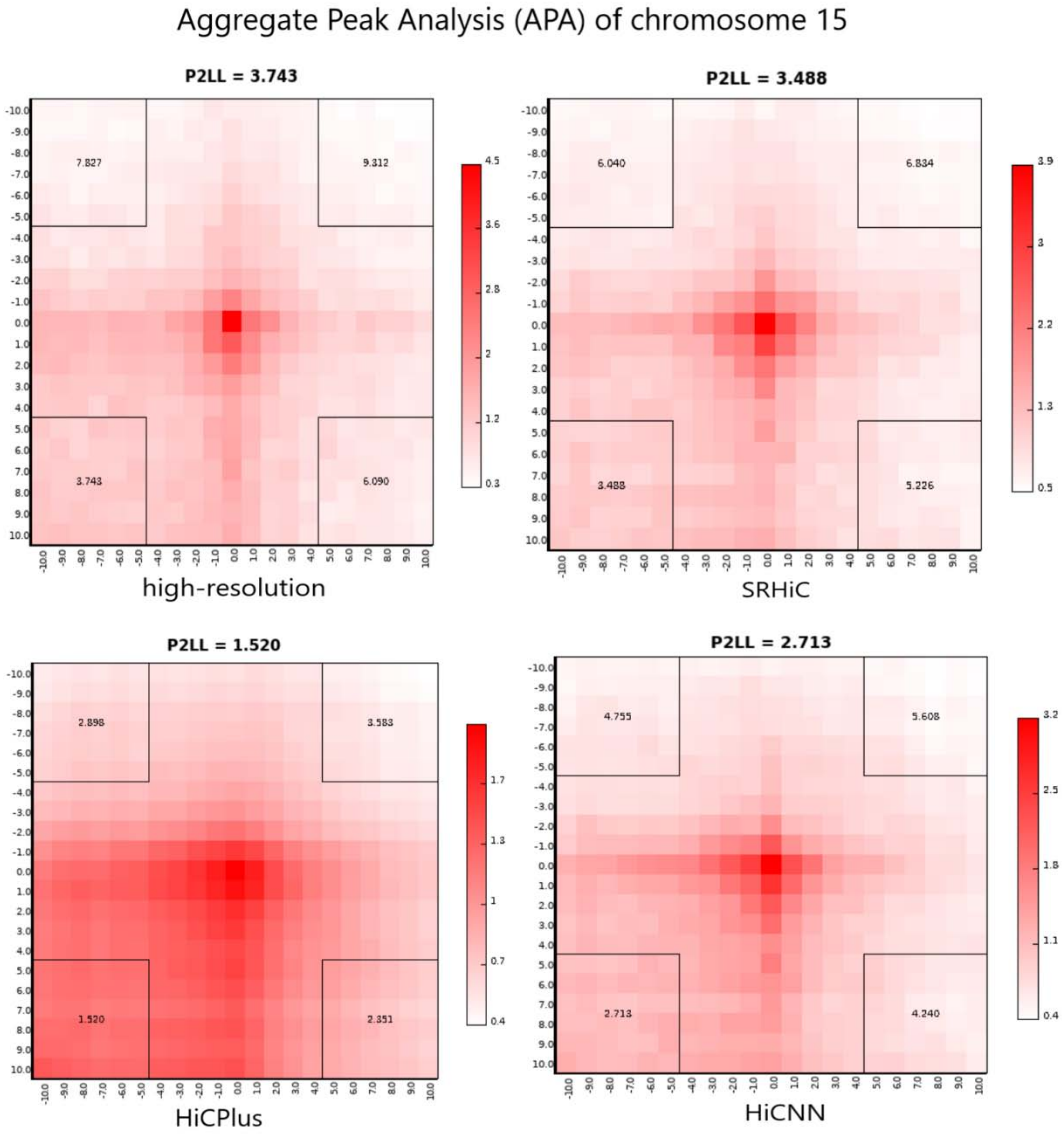

Figure S16. The Aggregate Peak Analysis (APA) of chromosome 15 in CH12-LX with down-sampled (1/16).

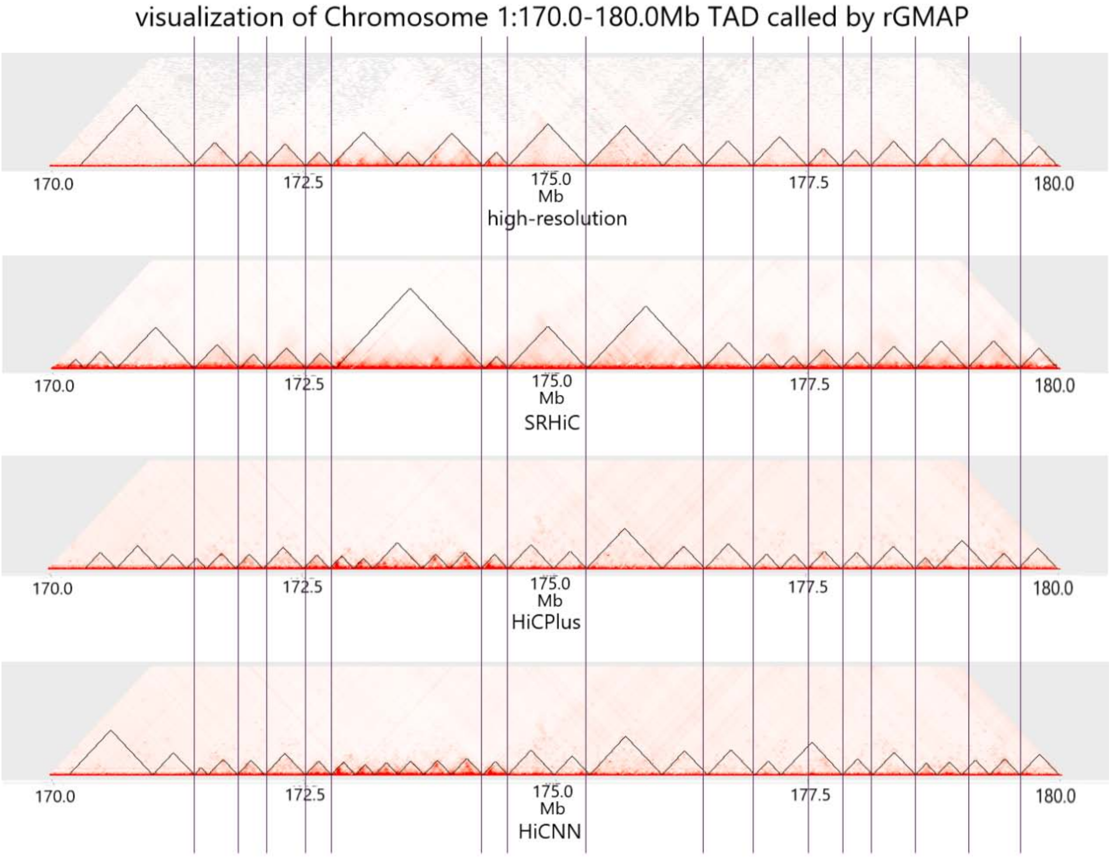

Figure S17. The TAD boundary visualization of Chromosome 1:170.0-180.0Mb with down-sampled (1/16) in CH12-LX.

Supplementary Table 1: Test set, validation set, training set and run time for different model training.

1. Predicting high-resolution Hi-C data from low-resolution Hi-C data with down-sample ratio 1/16

experiment number	test set (chromosome)	validation set (chromosome)	training set (chromosome)	model name	running time (s)
1	1,3,4	2	5~22	HiCPlus	962
				SRHiC	2369
				HiCNN	11801
2	5,6,8	7	1~4, 9~22	HiCPlus	869
				SRHiC	1748
				HiCNN	11711
3	9,10,12	11	1~8, 13~22	HiCPlus	680
				SRHiC	1876
				HiCNN	12498
4	13,15,16,17	14	1~12, 18~22	HiCPlus	568
				SRHiC	3061
				HiCNN	12689
5	18,19,20,22	21	1~17	HiCPlus	744
				SRHiC	2399
				HiCNN	15170

2. Predicting high-resolution Hi-C data from low-resolution Hi-C data with down-sample ratio 1/25

experiment number	test set (chromosome)	validation set (chromosome)	training set (chromosome)	model name	running time (s)
1	2,11,14,21	7	1, 3~6, 8~10, 12~13, 15~20, 22	HiCPlus	659
				SRHiC	1756
				HiCNN	15286

Supplementary Table 2:The numbers of statistically significant chromatin interactions identified by different models in GM12878. The numbers of overlap with statistically significant chromatin interactions in real high-resolution Hi-C data were also shown for each model. The down-sampled ratio is 1/16.

	**SRHiC-enhanced total**	**SRHiC-enhanced distance-range(50Kb-250Kb)**	**SRHiC-enhanced distance-range(250Kb-1Mb)**	**SRHiC-enhanced distance-range(1Mb-2Mb)**	**HiCPlus-enhanced total**	**HiCPlus-enhanced distance-range(50Kb-250Kb)**	**HiCPlus-enhanced distance-range(250Kb-1Mb)**	**HiCPlus-enhanced distance-range(1Mb-2Mb)**	**HiCNN-enhanced total**	**HiCNN-enhanced distance-range(50Kb-250Kb)**	**HiCNN-enhanced distance-range(250Kb-1Mb)**	**HiCNN-enhanced distance-range(1Mb-2Mb)**
**Chromosome 1**												
The numbers of identified chromatin interactions	157798	63167	61497	7669	212582	70571	93625	21299	178915	66869	74076	11694
Overlap with real high-resolution Hi-C data	121759	49587	46229	5761	128277	50567	50577	6789	126316	50627	48735	6576
**Chromosome 3**												
The numbers of identified chromatin interactions	108495	42233	44761	4166	147616	46275	68373	15052	120713	43183	52889	7379
Overlap with real high-resolution Hi-C data	91797	36413	36886	3126	98375	37069	41549	4300	95263	36761	39179	3991
**Chromosome 4**												
The numbers of identified chromatin interactions	78028	32668	29679	1529	114188	37657	51730	9844	91234	34725	38112	3961

Overlap with real high-resolution Hi-C data	65751	28133	24006	1119	72747	29274	28622	2129	70031	28954	26635	1814
**Chromosome 5**												
The numbers of identified chromatin interactions	104141	39830	42603	6114	128555	41285	58225	13331	120352	40012	54442	10263
Overlap with real high-resolution Hi-C data	82561	32399	32221	4493	85835	32292	34873	5211	85711	32164	34848	5211
**Chromosome 6**												
The numbers of identified chromatin interactions	117821	41998	52049	7944	138850	42817	65267	15085	131696	41798	62061	12260
Overlap with real high-resolution Hi-C data	94414	34701	40043	5952	96847	34521	42009	6660	96820	34432	42087	6662
**Chromosome 8**												
The numbers of identified chromatin interactions	85892	33015	34713	5548	105889	33993	47329	11785	99265	33359	43988	9220
Overlap with real high-resolution Hi-C data	68656	27174	26655	3886	71180	27029	28621	4536	71127	27061	28590	4512
**Chromosome 9**												
The	101	39847	42715	3786	129	42967	57418	12875	110	40943	47911	5427

numbers of identified chromatin interactions	838				449				217			
Overlap with real high-resolution Hi-C data	67708	26166	28712	2436	68787	26070	29455	2875	68323	26175	29097	2653
**Chromosome 10**												
The numbers of identified chromatin interactions	78688	30265	31210	5041	110496	34642	48092	14370	88164	31647	36963	6894
Overlap with real high-resolution Hi-C data	64609	25225	25034	3905	67423	24050	26435	2763	66928	25603	26302	4352
**Chromosome 12**												
The numbers of identified chromatin interactions	81507	31755	34120	3182	107914	33781	48467	12686	89042	32129	39373	4849
Overlap with real high-resolution Hi-C data	68563	27191	27938	2445	72480	27365	30689	3290	70400	27176	29240	2847
**Chromosome 13**												
The numbers of identified chromatin interactions	71892	27057	29682	3899	88743	28343	39536	9418	72322	26280	30485	4400
Overlap with real high-resolution Hi-C	48656	18629	19522	2504	48987	18487	19897	2638	48132	18446	19187	2518

data												
**Chromosome 15**												
The numbers of identified chromatin interactions	79570	31503	32802	3230	97787	32772	42453	9960	81143	30928	33830	4033
Overlap with real high-resolution Hi-C data	49091	19116	20694	1968	49520	19007	21033	2147	48900	19045	20523	1999
**Chromosome 16**												
The numbers of identified chromatin interactions	71448	27357	28445	5393	89413	28919	38430	11209	74273	27415	30047	6179
Overlap with real high-resolution Hi-C data	50892	20252	19242	3596	51922	20231	20051	3788	51019	20213	19309	3629
**Chromosome 17**												
The numbers of identified chromatin interactions	55374	22900	22451	1546	70576	23843	30688	6941	57099	22488	23513	2288
Overlap with real high-resolution Hi-C data	44401	18911	17337	1021	45968	18794	18459	1397	44444	18643	17347	1153
**Chromosome 18**												
The numbers of identified chromatin interactions	44808	17460	18498	1933	54580	17472	24465	5616	44702	16823	18474	2429

Overlap with real high-resolution Hi-C data	34804	13717	13872	1353	35373	13348	14429	1761	34293	13477	13513	1411
**Chromosome 19**												
The numbers of identified chromatin interactions	41047	17818	14754	1714	50717	18059	20073	5530	42095	17509	15434	2243
Overlap with real high-resolution Hi-C data	31269	14100	10419	1122	32091	13934	10925	1568	31282	14025	10349	1227
**Chromosome 20**												
The numbers of identified chromatin interactions	41449	16030	16794	2531	50481	15851	21906	6446	42092	15557	17310	3013
Overlap with real high-resolution Hi-C data	32244	12910	12638	1595	32812	12687	13082	1956	32101	12788	12498	1678
**Chromosome 22**												
The numbers of identified chromatin interactions	55397	19360	23196	5546	63766	20492	27782	7829	57529	20159	23925	5734
Overlap with real high-resolution Hi-C data	25415	8638	10617	2956	25141	8629	10545	2762	25220	8641	10521	2858

Supplementary Table 3: The numbers of active enhancer-promoter pairs overlap with annotation data identified by different models in GM12878.

	Overlap with active enhancer-promoter pairs in high-resolution Hi-C
down-sampling Hi-C	2
HiCPlus enhanced Hi-C	144
HiCNN enhanced Hi-C	146
SRHiC enhanced Hi-C	146

Supplementary Table 4: The numbers of statistically significant chromatin interactions identified by different models in GM12878. The numbers of overlap with statistically significant chromatin interactions in real high-resolution Hi-C data were also shown for each model. The down-sampled ratio is 1/25.

	**SRHiC-enhanced total**	**SRHiC-enhanced distance-range(50Kb-250Kb)**	**SRHiC-enhanced distance-range(250Kb-1Mb)**	**SRHiC-enhanced distance-range(1Mb-2Mb)**	**HiCPlus-enhanced**	**HiCPlus-enhanced distance-range(50Kb-250Kb)**	**HiCPlus-enhanced distance-range(250Kb-1Mb)**	**HiCPlus-enhanced distance-range(1Mb-2Mb)**	**HiCNN-enhanced**	**HiCNN-enhanced distance-range(50Kb-250Kb)**	**HiCNN-enhanced distance-range(250Kb-1Mb)**	**HiCNN-enhanced distance-range(1Mb-2Mb)**
**Chromosome 2**												
The numbers of identified chromatin interactions	128689	49269	53580	5460	184060	57740	84614	19485	136818	51621	57576	6355
Overlap with real high-resolution Hi-C data	105601	41632	42292	3690	113688	43595	47203	4269	107878	42638	43035	3756
**Chromosome 11**												
The numbers of identified chromatin interactions	84624	34713	34749	2155	123542	40171	53377	11136	90194	36222	37235	3305
Overlap with real high-resolution Hi-C data	70912	30082	27779	1366	76349	31515	30813	1733	72368	30751	28229	1445
**Chromosome 14**												
The numbers of identified chromatin interactions	56105	22116	22979	2400	99984	34492	43799	9067	65959	25934	27028	2973

Overlap with real high-resolution Hi-C data	46412	18871	18203	1675	53570	21515	21524	1945	49563	20279	19329	1732
**Chromosome 21**												
The numbers of identified chromatin interactions	25628	9184	9984	1856	46506	15909	20592	4417	30389	11386	12586	2102
Overlap with real high-resolution Hi-C data	19868	7737	7680	1358	23067	8894	9489	1380	21572	8457	8480	1377

Supplementary Table 5: The numbers of statistically significant chromatin interactions identified by different models in K562. The numbers of overlap with statistically significant chromatin interactions in real high-resolution Hi-C data were also shown for each model. The down-sampled ratio is 1/16.

	**SRHiC-enhanced total**	**SRHiC-enhanced distance-range(50Kb-250Kb)**	**SRHiC-enhanced distance-range(250Kb-1Mb)**	**SRHiC-enhanced distance-range(1Mb-2Mb)**	**HiCPlus-enhanced**	**HiCPlus-enhanced distance-range(50Kb-250Kb)**	**HiCPlus-enhanced distance-range(250Kb-1Mb)**	**HiCPlus-enhanced distance-range(1Mb-2Mb)**	**HiCNN-enhanced**	**HiCNN-enhanced distance-range(50Kb-250Kb)**	**HiCNN-enhanced distance-range(250Kb-1Mb)**	**HiCNN-enhanced distance-range(1Mb-2Mb)**
**Chromosome 1**												
The numbers of identified chromatin interactions	40596	19261	7610	504	70147	30732	20239	1837	64407	29557	16598	1057
Overlap with real high-resolution Hi-C data	30353	14115	5243	314	35832	16597	7012	358	36003	16757	7000	346
**Chromosome 2**												
The numbers of identified chromatin interactions	25049	10789	4629	152	40493	17320	11202	784	36098	15920	8845	337
Overlap with real high-resolution Hi-C data	18217	7567	3143	63	20749	8804	3985	87	20679	8777	3927	67
**Chromosome 3**												
The numbers of identified chromatin interactions	27782	12948	4985	147	39277	17793	10550	686	35909	16732	8640	322

Overlap with real high-resolution Hi-C data	20843	9327	3555	47	22594	10313	4232	71	22605	10306	4196	57
**Chromosome 4**												
The numbers of identified chromatin interactions	13885	5393	1662	61	23214	989	42	0	21383	9559	4070	133
Overlap with real high-resolution Hi-C data	9938	3657	1172	13	11730	927	42	0	11590	4607	1670	15
**Chromosome 5**												
The numbers of identified chromatin interactions	15862	7305	1615	164	28913	12560	7508	655	25552	11653	5515	273
Overlap with real high-resolution Hi-C data	12130	5434	1234	48	14664	6612	2201	69	14613	6603	2142	56
**Chromosome 6**												
The numbers of identified chromatin interactions	28390	13497	5540	497	43513	18834	13565	1494	39784	17933	11407	976
Overlap with real high-resolution Hi-C data	22012	10178	4161	312	24899	11380	5600	449	24923	11390	5590	469
**Chromosome 7**												

The numbers of identified chromatin interactions	25182	11308	5193	85	38939	16411	12064	969	35055	15177	10103	457
Overlap with real high-resolution Hi-C data	18840	8156	3597	52	21096	9210	4484	99	20996	9124	4510	89
**Chromosome 8**												
The numbers of identified chromatin interactions	18705	6894	2122	191	24069	10750	6180	599	21616	10042	4692	337
Overlap with real high-resolution Hi-C data	11103	4752	1433	101	12228	5480	1998	105	12235	5508	1960	90
**Chromosome 9**												
The numbers of identified chromatin interactions	18115	8254	3607	667	32476	14852	8187	892	30352	14217	6904	732
Overlap with real high-resolution Hi-C data	13397	5949	2503	585	15516	7110	3004	528	15563	7159	2983	543
**Chromosome 10**												
The numbers of identified chromatin interactions	19623	7422	3242	46	27052	11791	7952	848	24309	10848	6641	490
Overlap with real high-resolu	126	5580	2420	35	145	6522	3213	176	144	6478	3214	165

tion Hi-C data	77				29				71			
**Chromosome 11**												
The numbers of identified chromatin interactions	19286	9048	3175	63	29873	13425	8070	373	31066	12823	6524	141
Overlap with real high-resolution Hi-C data	14475	6525	2256	8	16610	7595	2991	16	16980	7641	2978	10
**Chromosome 12**												
The numbers of identified chromatin interactions	16457	7439	2786	142	25481	11149	6937	560	22986	10349	5501	299
Overlap with real high-resolution Hi-C data	12060	5305	1847	79	13605	6081	2391	88	13639	6080	2372	87
**Chromosome 13**												
The numbers of identified chromatin interactions	12574	3986	4637	1168	16090	6076	5011	666	15089	5720	4534	573
Overlap with real high-resolution Hi-C data	8907	3081	3110	580	8906	3434	2769	296	8996	3434	2832	303
**Chromosome 14**												
The numbers of identified chromatin	6758	2984	612	21	14325	6568	2589	92	12930	6022	1853	35

interactions												
Overlap with real high-resolution Hi-C data	4594	1958	406	3	5827	2570	632	7	5842	2607	605	5
**Chromosome 15**												
The numbers of identified chromatin interactions	7820	3561	1021	143	21794	9520	5633	382	19752	8986	4465	196
Overlap with real high-resolution Hi-C data	5675	2489	762	64	8074	3585	1472	83	8082	3596	1462	80
**Chromosome 16**												
The numbers of identified chromatin interactions	11748	5577	1829	411	22686	10096	5807	689	20610	9452	4714	472
Overlap with real high-resolution Hi-C data	8597	3929	1279	201	10460	4776	1765	245	10456	4785	1756	246
**Chromosome 17**												
The numbers of identified chromatin interactions	9847	4842	1529	43	17217	7766	4686	255	15340	7254	3528	96
Overlap with real high-resolution Hi-C data	7681	3576	1103	8	9395	4295	1523	19	9352	4277	1490	10
**Chromosome 18**												

The numbers of identified chromatin interactions	8336	3905	1208	137	13071	6083	3206	289	11813	5656	2490	165
Overlap with real high-resolution Hi-C data	6021	2674	839	41	6754	3112	1076	46	6780	3101	1083	48
**Chromosome 19**												
The numbers of identified chromatin interactions	5931	2957	515	50	11673	5199	2840	211	10095	4792	1920	109
Overlap with real high-resolution Hi-C data	4331	2119	349	19	5545	2652	652	25	5482	2666	625	23
**Chromosome 20**												
The numbers of identified chromatin interactions	9058	4359	1466	62	14636	6507	4084	287	13138	6120	3208	159
Overlap with real high-resolution Hi-C data	6615	3098	1019	28	7639	3558	1371	49	7580	3541	1361	42
**Chromosome 21**												
The numbers of identified chromatin interactions	9209	3843	2200	401	16895	7549	4756	592	16047	7359	4236	478
Overlap with real high-resolu	626	2592	1328	247	728	3197	1611	264	737	3241	1624	280

tion Hi-C data	7				4				1			
**Chromosome 22**												
The numbers of identified chromatin interactions	234<br>77	6147	12362	1584	239<br>84	7306	12440	2242	236<br>68	7231	12306	2193
Overlap with real high-resolution Hi-C data	190<br>85	5667	10000	1031	181<br>74	5808	9571	1379	183<br>02	5810	9671	1403

Supplementary Table 6: The numbers of active enhancer-promoter pairs overlap with annotation data identified by different models in K562.

	Overlap with active enhancer-promoter pairs in high-resolution Hi-C
high-resolution Hi-C	151
HiCPlus enhanced Hi-C	112
HiCNN enhanced Hi-C	114
SRHiC enhanced Hi-C	115

Supplementary Table 7: The numbers of statistically significant chromatin interactions identified by different models in CH12-LX. The numbers of overlap with statistically significant chromatin interactions in real high-resolution Hi-C data were also shown for each model. The down-sampled ratio is 1/16.

	**SRHiC-enhanced total**	**SRHiC-enhanced distance-range(50Kb-250Kb)**	**SRHiC-enhanced distance-range(250Kb-1Mb)**	**SRHiC-enhanced distance-range(1Mb-2Mb)**	**HiCPlus-enhanced**	**HiCPlus-enhanced distance-range(50Kb-250Kb)**	**HiCPlus-enhanced distance-range(250Kb-1Mb)**	**HiCPlus-enhanced distance-range(1Mb-2Mb)**	**HiCNN-enhanced**	**HiCNN-enhanced distance-range(50Kb-250Kb)**	**HiCNN-enhanced distance-range(250Kb-1Mb)**	**HiCNN-enhanced distance-range(1Mb-2Mb)**
**Chromosome 1**												
The numbers of identified chromatin interactions	10668	3965	834	14	17711	8158	2172	37	17040	8017	1595	21
Overlap with real high-resolution Hi-C data	8267	3124	623	5	10996	4790	886	8	11190	4932	832	7
**Chromosome 2**												
The numbers of identified chromatin interactions	11568	4137	1086	12	20075	9622	2541	30	18803	9053	1873	16
Overlap with real high-resolution Hi-C data	8905	3271	763	1	12098	5370	1008	1	12118	5403	950	1
**Chromosome 3**												
The numbers of identified chromatin interactions	11127	4391	808	13	15002	6707	1919	37	14543	6789	1428	18

Overlap with real high-resolution Hi-C data	7637	2943	553	2	9415	3993	737	3	9618	4193	712	2
**Chromosome 4**												
The numbers of identified chromatin interactions	9821	3711	420	16	16729	8050	1610	38	16074	7901	1160	19
Overlap with real high-resolution Hi-C data	7525	2894	299	5	10277	4626	516	6	10323	4719	471	5
**Chromosome 5**												
The numbers of identified chromatin interactions	8918	2792	853	7	15811	7111	1905	36	15083	6939	1441	20
Overlap with real high-resolution Hi-C data	6841	2220	599	5	9499	3907	716	13	9583	3987	699	11
**Chromosome 6**												
The numbers of identified chromatin interactions	8084	2579	719	21	13964	6260	1842	34	13188	6114	1399	10
Overlap with real high-resolution Hi-C data	6134	2025	503	15	8458	3477	745	4	8510	3573	717	3
**Chromosome 7**												

The numbers of identified chromatin interactions	9991	3618	728	83	18461	8931	1784	52	17527	8620	1330	47
Overlap with real high-resolution Hi-C data	7644	2840	504	41	10867	4862	694	26	10853	4917	656	25
**Chromosome 8**												
The numbers of identified chromatin interactions	9021	3397	916	12	14711	6890	1916	37	14107	6758	1560	22
Overlap with real high-resolution Hi-C data	6922	2613	638	9	9190	3971	844	9	9272	4070	819	8
**Chromosome 9**												
The numbers of identified chromatin interactions	6670	5841	1297	5	13123	6261	1887	19	12240	6077	1390	5
Overlap with real high-resolution Hi-C data	5169	3189	578	1	7620	3499	672	1	7617	3569	641	1
**Chromosome 10**												
The numbers of identified chromatin interactions	7258	2463	917	7	12423	5664	1848	19	11726	5517	1408	11
Overlap with real high-resolu	553	1945	652	2	740	3135	777	2	745	3236	735	2

tion Hi-C data	2				1				0			
**Chromosome 11**												
The numbers of identified chromatin interactions	13014	5852	1635	27	23632	12094	4150	83	21960	11622	3214	34
Overlap with real high-resolution Hi-C data	10283	4772	1138	4	14325	7292	1536	7	14281	7354	1504	4
**Chromosome 12**												
The numbers of identified chromatin interactions	6986	4933	1032	17	13753	5395	1597	28	11439	5257	1214	19
Overlap with real high-resolution Hi-C data	5322	2986	578	7	7205	3054	667	6	7240	3111	607	9
**Chromosome 13**												
The numbers of identified chromatin interactions	4856	1324	794	10	9498	3903	1648	49	8804	3753	1210	21
Overlap with real high-resolution Hi-C data	3659	1036	569	5	5209	1922	693	15	5252	1974	656	6
**Chromosome 14**												
The numbers of identified chromatin	9673	3838	779	0	14762	6885	1922	27	14150	6895	1450	13

interactions												
Overlap with real high-resolution Hi-C data	7224	2921	568	0	9127	4118	764	1	9181	4215	726	0
**Chromosome 15**												
The numbers of identified chromatin interactions	10894	4768	1374	27	16127	8045	2513	69	15363	7850	2053	30
Overlap with real high-resolution Hi-C data	8470	3702	1019	19	10503	5007	1189	19	10567	5084	1184	13
**Chromosome 16**												
The numbers of identified chromatin interactions	12220	4899	2231	45	17210	8117	3380	75	16201	7908	2782	54
Overlap with real high-resolution Hi-C data	9318	3769	1611	31	11034	4998	1656	33	11056	5088	1635	29
**Chromosome 17**												
The numbers of identified chromatin interactions	7124	2606	691	20	11827	5620	1496	29	11239	5386	1171	17
Overlap with real high-resolution Hi-C data	5541	2003	527	12	7352	3183	664	13	7436	3244	656	13
**Chromosome 18**												

The numbers of identified chromatin interactions	3655	1316	283	0	7026	3187	918	11	6562	3086	601	5
Overlap with real high-resolution Hi-C data	2757	1044	184	0	4030	1772	304	0	4048	1813	284	0
**Chromosome 19**												
The numbers of identified chromatin interactions	3072	1099	210	0	7195	3565	913	11	6605	3318	660	5
Overlap with real high-resolution Hi-C data	2458	918	153	0	3962	1848	296	2	3912	1840	269	1
